# Background Strain and the Differential Susceptibility of Podocyte-Specific Deletion of *Myh9* on Murine Models of Experimental Glomerulosclerosis and HIV Nephropathy

**DOI:** 10.1371/journal.pone.0067839

**Published:** 2013-07-10

**Authors:** Duncan B. Johnstone, Omer Ikizler, Jidong Zhang, Lawrence B. Holzman

**Affiliations:** 1 Division of Nephrology, Temple University School of Medicine, Philadelphia, Pennsylvania, United States of America; 2 Renal-Electrolyte and Hypertension Division, University of Pennsylvania School of Medicine, Philadelphia, Pennsylvania, United States of America; 3 Philadelphia VA Medical Center, Philadelphia, Pennsylvania, United States of America; University of Houston, United States of America

## Abstract

We previously reported that podocyte-specific deletion of *Myh9* (conventional myosin heavy chain 2A) in C57BL/6 mice does not cause spontaneous kidney disease but instead results in a predisposition to glomerulosclerosis in response to a second model of glomerular injury. In contrast, other investigators reported that podocyte-specific deletion of *Myh9* (*Pod*Δ*Myh9*) resulted in spontaneous glomerulosclerosis in mice on a mixed background, suggesting that the glomerulosclerosis is dependent on background strain. In order to elucidate the cause of this strain dependent effect *Podocin::Cre* and *Myh9^flox^* alleles were backcrossed to mouse strain FVB/N, which is highly susceptible to glomerulosclerosis, with the aim of intercrossing susceptible FVB/N and resistant C57BL/6 mice in subsequent congenic analyses. However, after backcrossing mice to FVB/N and aging mice to 28 weeks, we found no evidence of glomerular disease in PodΔ*Myh9* mice vs control littermates (urine MAC ratio all p>0.05). We also tested C57BL/6 PodΔ*Myh9* mice for a predisposition to injury from models other than Adriamycin including HIV nephropathy (HIVAN), puromycin nephropathy, and sheep nephrotoxic serum. In the *Tg26* model of HIVAN, we found that podocyte-specific deletion of Myh9 resulted in a modest hypersensitivity in adults compared to Tg26+ control littermates (urine MAC ratio, p<0.05 or less). In contrast, we found that PodΔ*Myh9* mice were not predisposed to injury in response to other injury models including puromycin nephropathy and sheep nephrotoxic serum. While the mechanism of injury in these models is not fully understood, we conclude that PodΔ*Myh9* results in a variable susceptibility to glomerulosclerosis in response to different models of glomerular injury. In addition, based on the lack of a spontaneous phenotype of glomerulosclerosis in both C57BL/6 and FVB/N mice, we propose that *Myh9* is not absolutely required in adult podocytes.

## Introduction

Sequence variants in *MYH9* encoding myosin heavy chain 2A have been associated with two forms of kidney disease. The foremost is a rare, autosomal dominant form of focal and segmental glomerulosclerosis (FSGS) due to “*MYH9*-related disease,” a term uniting four previously distinct Giant Platelet Syndromes: May-Hegglin Anomaly, Sebastian Syndrome, Epstein's Syndrome and Fetchner's Syndrome, all of which result from dominant mutations in *MYH9* [1]. One-third of patients with dominant *MYH9*-mutations, those previously classified as having Epstein's or Fetchner's Syndrome, develop glomerulosclerosis and progress to end stage kidney disease requiring dialysis or transplantation by the 2^nd^ or 3^rd^ decade [1], [2]. While glomerulosclerosis arises more often in patients with missense mutations that affect the N-terminal motor domain of *MYH9* rather than the tail domain, patients with the same amino acid change can develop any of the four previously distinct syndromes [1], [3]. The mechanism for the apparent all-or-nothing phenotype of glomerulosclerosis from patients with the same dominantly inherited amino acid change in MYH9 is not understood.

A second form of kidney disease attributed to *MYH9* is a possible predisposition to common forms of kidney disease, an idea that arose from initial reports that *MYH9* polymorphisms in several introns that comprise the E1, F1 and S1 haplotypes of *MYH9* are associated with both primary and HIV-related glomerulosclerosis, non-diabetic ESRD, and hypertensive nephrosclerosis in African-Americans and Hispanic-Americans [4]–[7]. Subsequent research demonstrated that a majority of the signal at *MYH9* among African-Americans resulted from linkage to polymorphisms in the neighboring gene *APOL1* [8], [9]. Nevertheless, additional studies continue to report a correlation between intronic *MYH9* polymorphisms and kidney disease in many contexts including a persistent signal (albeit smaller) for *MYH9* among African-Americans with FSGS that remains after controlling for the *APOL1* G1 and G2 alleles [10], a modest association of sickle cell nephropathy with one separate and independent polymorphism each in *MYH9* and *APOL1* [11], a modest association of intronic *MYH9* polymorphisms with diabetic nephropathy among European-Americans [12] the association of one *MYH9* polymorphism with non-diabetic kidney disease in European Americans [13], and no association of IgA nephropathy with polymorphisms in either *MYH9* or *APOL1* [14]. For lupus nephritis, the complicated findings include a small correlation between nephritis and several intronic *MYH9* polymorphisms among European-Americans [15], a correlation with a different *MYH9* polymorphism among the Gullah [15], and no correlation with lupus nephritis and polymorphisms in either *MYH9* or *APOL1* among African-Americans [15], [16].

Why *MYH9* intronic polymorphisms would be associated with some but not all common kidney diseases remains unclear, as does the underlying mechanism by which *MYH9* autosomal dominant mutations result in glomerulosclerosis. In order to improve our understanding of these phenomena we have examined *MYH9*-related kidney disease in murine models. A classical knockout of *Myh9* in mice is uninformative because mice die early in embryonic development at E6.5 during gastrulation [17]. In our previous work, based on the phenotype of glomerulosclerosis in patients with both Epstein's or Fetchner's syndrome and the association of risk polymorphisms in *MYH9* with idiopathic and HIV-related glomerulosclerosis, we hypothesized that a predominant site of action should be the podocyte. Based on the role of conventional Myosin in cellular functions that are of particular importance to podocyte cell biology including cytoskeletal tensile strength and cell-cell adhesion [18], our expectation was that mice with a podocyte-specific deletion of *Myh9* (PodΔ*Myh9)* would develop severe perinatal glomerular disease. To our surprise, PodΔ*Myh9* mice on the C57BL/6 background reached weaning at the expected Mendelian frequency and had no evidence of glomerulosclerosis compared to control littermates based on growth, albuminuria, blood urea nitrogen, serum creatinine or histopathology, despite aging until 9 months [3]. Secondly, we found that these PodΔ*Myh9* mice were hypersensitive to podocyte injury. When challenged with Adriamycin they developed significant glomerulosclerosis in comparison to control littermates on the C57BL/6 background strain [3].

In contrast, on a mixed strain background, other investigators found that PodΔ*Myh9* does result in spontaneous glomerulosclerosis. Investigators at the NIH found that roughly 30% of PodΔ*Myh9* mice on a mixed background of strains C57BL/6, BALB/c, and 129/SJ developed severe albuminuria and a histopathology resembling focal and segmental glomerulosclerosis [19]. (and Dr. R.S. Adelstein, personal communication). The difference in these two findings suggested an effect of mouse background strain. In mice, several examples of strain-specific kidney disease have been described in which the C57BL/6 strain is generally resistant to experimental glomerular disease, whereas the BALB/c and FVB strains are sensitive to glomerular disease including Adriamycin nephropathy [20], HIV-nephropathy [21], puromycin aminonucleoside [22], the tetraspannin mutant CD151 [23] and others.

In this report we have extended our analysis of *Myh9* function in the podocyte *in vivo* in two areas: first, if PodΔ*Myh9* on the C57BL/6 background has no overt phenotype but the same deletion on a mixed background results in spontaneous and severe glomerulosclerosis, we hypothesized that mouse congenics could identify the genetic loci responsible for this strain specific resistance or sensitivity to PodΔ*Myh9*. Mouse congenics has elegantly uncovered genes responsible for the strain-specific predisposition to Adriamycin nephropathy [20] and HIV-nephropathy [21], providing insight into the basic mechanisms underlying these two forms of glomerular injury. Furthermore, in a comprehensive analysis of the differential sensitivity to HIV nephropathy among mouse strains, a phylogenetic tree of laboratory mouse strain correlated closely with the susceptibility of strains to HIV nephropathy; strains related to C57BL/6 were resistant to HIV nephropathy and shared a gene regulatory network that included increased expression of *Nphs2* (Podocin), while strains genetically related to BALB/c and FVB showed a distinct gene regulatory network and were sensitive to experimental HIV nephropathy [21]. We reasoned that elucidation of the genetic loci underlying strain-specific effects of PodΔ*Myh9* on kidney disease in mice might be extrapolated to humans. For instance, transgenic mouse models with missense mutations that are orthologous to human Epstein and Fetchner's syndrome have recently been described, and in addition to recapitulating the phenotype of thrombocytopenia and Giant Platelet size these mice also develop progressive glomerulosclerosis [19]. In human patients with autosomal dominant *MYH9*-disease some families with the identical mutation and similar platelet phenotypes have an all-or-nothing kidney disease, suggesting that additional gene-gene or gene-environment interactions are involved in *MYH9*-dependent glomerulosclerosis [3]. The second area we addressed was whether podocyte-specific deletion of *Myh9* on the C57BL/6 background results in a general susceptibility to all forms of experimental glomerular injury (a “weakened podocyte” effect), or whether the hypersensitivity is specific to Adriamycin nephropathy, which could arise through shared pathophysiology.

To our surprise, after backcrossing the *Podocin::Cre* and *Myh9^flox^* alleles from the C57BL/6 strain, which is generally resistant to glomerular disease, onto the FVB/N strain, which is generally sensitive to glomerular disease, we found no evidence of glomerulosclerosis in PodΔ*Myh9* adult mice. In our second inquiry, we found that PodΔ*Myh9* on the C57BL/6 background results in a modest hypersensitivity to experimental HIV-nephropathy but no hypersensitivity to several other models of experimental glomerulosclerosis including puromycin aminonucleoside and sheep nephrotoxic serum.

## Results

We backcrossed the *Podocin::Cre* and *Myh9^flox^* alleles from the C57BL/6 background onto the FVB/N background, which is more sensitive to many forms of experimental glomerular disease as described above. In work by colleagues described above, we hypothesized that glomerulosclerosis was present in 30% of mice on a mixed background due to genetic mixing of sensitivity and resistance factors from the 3 strains employed; a single strain with a proclivity to experimental kidney disease, we reasoned, might result in a more highly penetrant phenotype that would be more amenable to congenic analysis. During the first four generations, we accelerated the pace of backcrossing through the use of speed congenics (Max-Bax), which is based on the use of polymorphisms that are evenly spaced along each chromosome and differ between the C57BL/6 and FVB/N strains. After the 4^th^ generation, we chose a founder double heterozygous mouse that was 96.53% FVB (Fig. S1), and after two more generations of backcrossing, we began our experiments with N6 mice that were approximately 99.3125% FVB.

**Figure 1:**
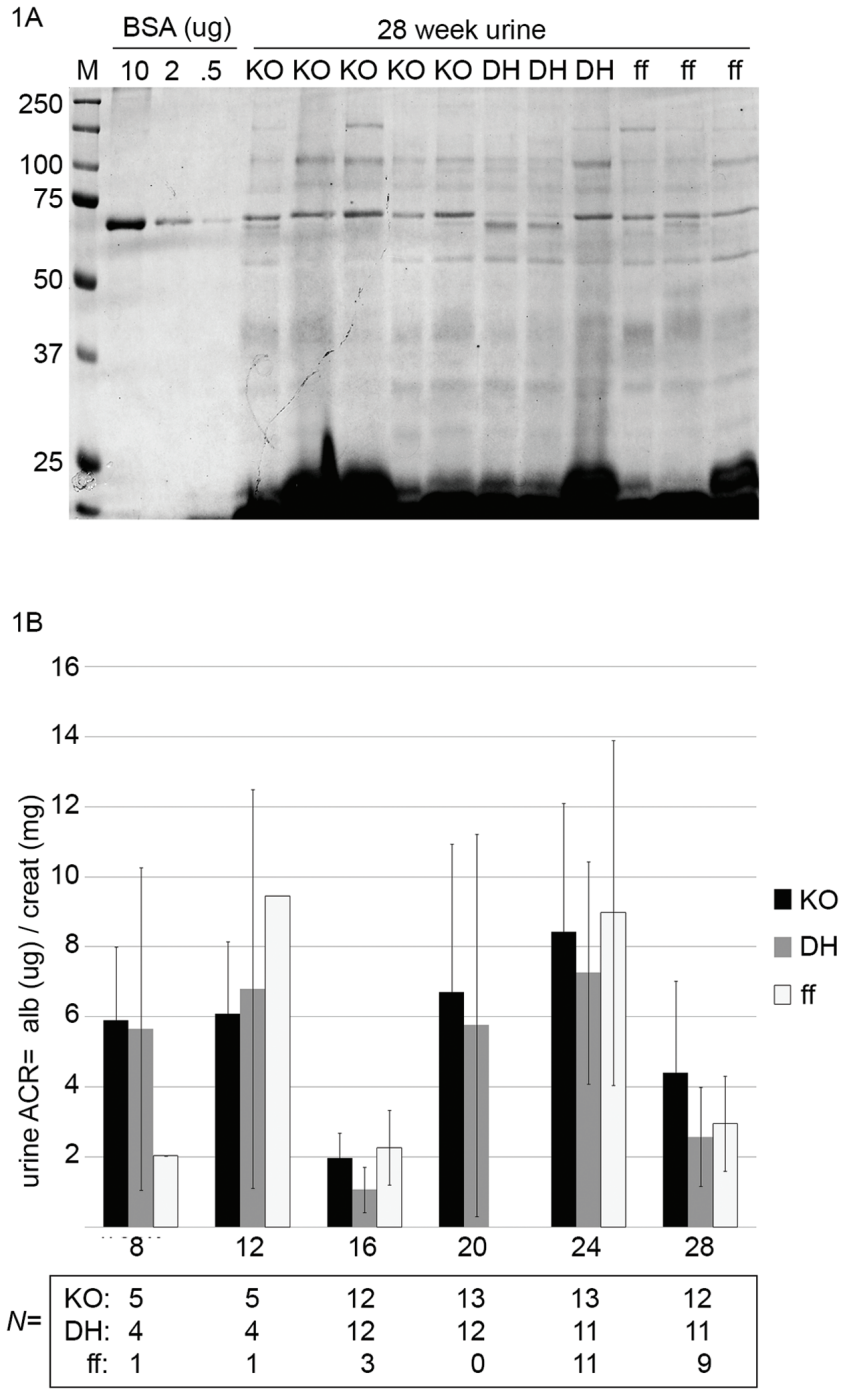
Podocyte-specific knockout of ***Myh9*** **does not result in progressive albuminuria on the FVB mouse strain background.** (A) Urine from all mice was screened for albuminuria every 4 weeks by SDS-PAGE with Coomassie stain. Shown here is one of 2 gels from 28 week old mice. M = marker (Precision-Plus Dual Color, Bio-Rad), BSA = bovine serum albumin diluted from 1 mg/mL stock to the indicated amounts per lane (ug). Sample lanes contain 10 uL of mouse urine of the indicated genotype: (KO) = *Podocin::Cre*/*+*; *Myh9^flox^*/*^flox^*, (DH) = *Podocin::Cre*/*+*; *Myh9^flox^*/*+*, (FF) = *cre−; Myh9^flox^*/*^flox^*. As an internal control, we observed low molecular weight (tubular) proteinuria from each urine sample lane but no significant albuminuria at this time or at any other time point. These urine samples are not normalized for concentration but provide an initial screen for albuminuria. (B) Quantification of albuminuria by urine albumin::creatinine ratio (ACR). Albuminuria was measured by plate ELISA (albuwell-M kit, Exocell/Glycadia) and creatinine was measured by endpoint-assay (Teco Diagnostics). For both assays, every plate included 8 standards and only plates for which the standards fit a straight line (r^2^>0.98) were accepted for analysis. All results from mouse urine that fell outside the linear range were repeated using additional urine dilutions. Legend = mouse genotypes as described above. Y-axis = urine to creatinine ratio (in ug/mg) +/− standard deviation. X-axis = urine collections every four weeks from week 8 onwards. The table below the X axis shows the number of animals (*N*) assayed at each timepoint. Urine samples of different genotypes but from the same month were tested in all possible cases on the same ELISA plate. There were no statistically significant differences by ANOVA (In-Stat3) for all groups and all time points with the exception of some 16 week and 28 week time points, which fit a pattern suggestive of plate-plate variation rather than progressive glomerulosclerosis. For instance, the 16 week KO is significantly lower than the KO at 24 weeks (by ANOVA with Tukey-Kramer post test <0.001), which could be interpreted as progression of disease in the KO, but in truth the 16 week DH control is also significantly lower than the KO at 24 weeks (by ANOVA with Tukey-Kramer post-test of p<0.001), and the 16 week values that are significantly lower than their counterparts at 24 weeks are not significantly different from each other (the 16 week KO vs. 16 week DH control or the 16 week ff control are not significantly different), and the values at 24 weeks for all three genotypes are not significantly different from each other (P>0.05 for all combinations of 24 week KO versus DH versus ff). It is simply that all 16 week values were atypically low, due either to aberrancy in the ELISA plate or to an unknown confounder at the time of that urine collection. Similarly, the 28 week DH and ff groups are significantly different than the KO 24 week time point (p<0.05 by Tukey-Kramer), but this is also true of the ff 24 week time point (p<0.05 by Tukey Kramer), and in both cases the 28 week time point that is different is showing less albuminuria than the 24 week time point, and it is the DH and FF controls that are different at 28 weeks, not the KO. Among the many groups compared by ANOVA, a few (20 wk DH, 24 wk DH and 28 wk DH) do not pass the Kolmogorov and Smirnov analyses for a normal distribution, but when post-test comparisons with these groups are analyzed by non-parametric Dunn's, there is no change in which groups demonstrate a statistically significant difference. In summary, based on multiple comparisons between groups, the urine ACR of podocyte-specific *Myh9* KO mice is not significantly different from control littermates within the same month, and podocyte-specific *Myh9* KO mice do not show progressive albuminuria with aging from 8 weeks to 28 weeks.

From experimental crosses of N6 double heterozygous animals (*Podocin::Cre/+*; *Myh9^flox^*/+)×N6 *Myh9^flox/flox^* mice, four genotypes were obtained at ¼ frequency each as outlined in Table 1, in which both podocyte knockout (PodΔ*Myh9*, also called “KO” due to space limitations in some figures) and DHet control mice inherited a single allele of *Podocin::Cre* as shown. Tail genotyping at weaning revealed the 4 genotypes were present at the expected Mendelian frequency (*N* of 28, 35, 28 and 29 weanlings respectively, Chi test = 0.89) indicating that the KO had no overt effect on development up to this age. Urine was collected from these mice every 4 weeks for 28 weeks and screened for albuminuria by Coomassie-stained PAGE. For all time points there was no gross difference in albuminuria in KO animals compared with control littermates (Fig. 1A). Moreover, quantitative assessments of albuminuria by plate ELISA demonstrated no significant difference in the urine albumin-to-creatinine ratio (ACR) of KO mice compared to control littermates on the FVB/N background, despite aging to 28 weeks (Fig. 1B).

**Table 1:** Expected genotypes of PodΔ*Myh9* and littermates as generated in this study from parents^a^ of genotype *Pod::Cre/+*; *Myh9^flox^*/+×N6 *Myh9^flox/flox^*.

Cohort of littermates	Genotype	Expected (and observed) frequency
“KO” or PodΔ*Myh9*	*Pod::Cre/+*; *Myh9^flox^*/*^flox^*	¼
“DHet” control	*Pod::Cre/+*; *Myh9^flox^*/*^+^*	¼
“FF” control	+/+ (cre−) ; *Myh9^flox^*/*^flox^*	¼
Not used	+/+ (cre−) ; *Myh9^flox^*/*^+^*	¼

a each parent was backcrossed first to >99% FVB/N.

**Figure 2:**
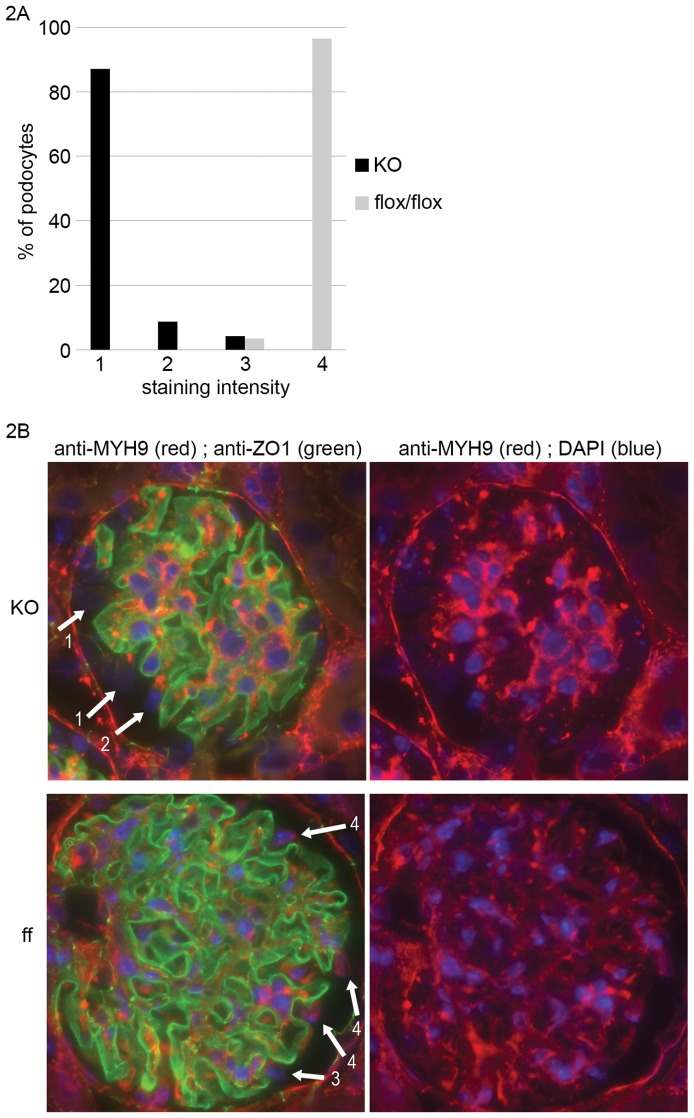
MYH9 podocyte staining verifies deletion in PodΔ***Myh9*** **mice.** MYH9 protein was assessed by immunofluoresence in perfusion-fixed, paraffin-embedded kidneys of adult mice. Within the glomerulus it can be difficult to identify podocytes from mesangial cells or the endothelium, and in the KO mice, Myh9 staining of mesangial cells towards the center of the glomerulus remains quite bright. Definitive podocytes can be assessed by considering cells that are on the outside of green ZO-1 staining and are facing into Bowman's space. Arrows in (B) point to several podocytes, and in the KO the nuclei remain visible with DAPI, but the diffuse red cytoplasmic staining of Myh9 is absent. (A) Tally of podocyte staining from 178 podocytes of 35 glomeruli from 2 KO mice (*Podocin::Cre*/+; *Myh9^flox^*/*^flox^*) compared to 114 podocytes of 21 glomeruli from “ff control” littermates (*cre−*; *Myh9^flox^*/*^flox^*). Scoring of MYH9 staining intensity was subjective, based on agreement of 2 individuals: (1) = no staining, (2) = faint/equivocal staining, (3) = moderate/incomplete staining, (4) = robust staining. The majority of podocytes from KO mice scored a “1” (no staining) and the majority of podocytes from control ff mice scored a “4” (robust staining). There is a significant difference between the KO and ff control MYH9 staining intensity in podocytes (non parametric Mann-Whitney Rank Sum test, p<0.0001). (B) Examples of stained glomeruli used in scoring from part (A), taken at 630× magnification from KO mice (top) and ff control mice (bottom) with representative podocytes and subjective scores (arrows with numbers 1–4). To minimize differences in processing and staining between genotypes, deparaffinization, blocking, staining and scoring were performed on kidneys of each genotype in parallel. Two primary antibodies were mixed together (rabbit α-MYH9 and mouse α-ZO1) as a “master mix” then aliquoted to all sections (except a secondary-only control). Both secondary antibodies were mixed together (Alexa-Fluor, 1∶1500 goat α-mouse488 and 1∶1500 goat α-rabbit594 then aliquoted to all sections. As seen in Fig. 2B, ZO-1 staining was similar in KO and ff control mouse glomeruli suggesting that the steps of deparafinization through antibody washing were performed consistently on slices handled in parallel, and this was also true for MYH9 staining of tubules and of mesangial cells. Occassional podocytes from control mice showed reduced staining, “3,” which we speculate could arise from podocytes that were obliquely cut such that only a small portion of cytoplasm was included on the slice. As previously reported we speculate that the few punctae of staining in our KO mice may represent inclusion bodies [3], but diffuse cytoplasmic staining as seen in control mice was never observed in KO mouse podocytes. The absence of robust staining (“4”) and the minimal moderate/equivocal staining (“3”) from 178 KO mouse podocytes suggests that mosaicism with incomplete gene excision occurred at a very low level in our KO mice. While we have not observed a difference in staining intensity between the *Myh9^flox^*/*^flox^* allele and the *Myh9* wild type allele, floxed alleles can theoretically reduce expression, which prompted us to choose the *Myh9^flox^* ^/*flox*^ mice rather than wild type mice as controls for staining in parallel with the KO mice.

As the lack of phenotype ran contrary to our expectations, we performed immunostaining to confirm that, after backcrossing to the FVB/N background, the Podocin::Cre transgene deleted *Myh9* from podocytes with high efficiency as previously demonstrated on the C57BL/6 background [3]. In our FVB cohort we found reproducible and specific staining of MYH9 protein in podocyte cytoplasm. Compared to control littermates that were deparrafinized and stained by immunofluoresence in parallel, kidney sections from KO mice revealed similarly robust MYH9 immunofluoresence in tubules and the mesangium but absent staining in podocytes (Fig. 2B.) To quantify this difference in podocyte staining, we subjectively scored all podocytes from randomly selected glomeruli in KO versus control animals, limiting our tally to cells on the outside of glomerular loops within Bowman's space that could clearly be identified as visceral glomerular epithelia (podocytes). Comparing over 100 podocytes from two KO mice versus Cre-negative *Myh9^flox/flox^* control mice, we found a marked difference in the staining intensity of MYH9 protein in podocytes (Fig. 2A). These results indicate that Cre-mediated excision of the *Myh9^flox^*.allele occurred efficiently on the FVB/N strain background. Cre-mediated mosaicism or incomplete excision could result in intermediate staining intensity or variably robust staining in rare podocytes in KO kidneys, but we found that 0% of 178 podocytes showed robust staining, 4% showed modest staining, and 8% showed faint staining, while the remainder had no cytoplasmic staining (Fig. 2A). At the level of our immunodetection, these results demonstrate a minimal amount of mosaicism, which is insufficient to explain the lack of a phenotype. We conclude that PodΔ*Myh9* does not result in spontaneous glomerulosclerosis on the FVB/N background, even after aging to 28 weeks. Alternative explanations for the difference in our findings compared with our colleagues from NIH are speculatory and are detailed in the Discussion.

To further explore our previous finding of a predisposition of to glomerulosclerosis in PodΔ*Myh9* mice on the C57BL/6 background in response to Adriamycin, we tested whether these mice are predisposed to glomerulosclerosis in response to other models of glomerular injury. We first examined the well-described *Tg26*-transgenic model of experimental HIV nephropathy [24]. The *Tg26* transgene includes a partial HIV proviral DNA including *Nef*, Vpr and *Tat*, which are the principal mediators of HIV-nephropathy; the transgene is deleted for *Gag* and *Pol*, rendering it non-infectious [25]. On the FVB/N background, *Tg26* causes severe nephropathy (death by 6–12 weeks in heterozygotes, and rare survival to weaning in homozygotes), while on the C57BL/6 background there is no kidney phenotype [26]. After backcrossing *Tg26* to C57BL/6 for six generations, pilot experiments demonstrated no significant difference in albuminuria in triple knockout mice compared to *Tg26*+ control littermates (defined below) at 6 or 8 weeks of age (Fig. S2). At seven generations of backcrossing we generated larger experimental cohorts by crossing N6 triple heterozygous “THet” males X N10 *Myh9^flox/flox^* females with the expectation of 8 genotypes of 7× backcrossed offspring as outlined in Table 2, including the triple knockout “TKO” (*Tg26/+*; *Podocin::Cre*/+; *Myh9^flox/flox^*), the triple heterozygous control “THet” (*Tg26/+*; *Podocin::Cre*/+; *Myh9^flox^*/+), and the “Tg26” control (*Tg26/+*; +/+; *Myh9^flox^*/+). As previously reported for the C57BL/6 background, we expected the “Tg26” control to have no phenotype. We reasoned that the THet controls might have an intermediate phenotype due to haploinsufficiency or loss of heterozygosity, but these THets control for the possibility of synergistic toxicity of the Cre transgene.

**Table 2:** Expected genotypes of *Tg26* PodΔ*Myh9* triple KO mice and control littermates as generated in this study from parents^d^ of genotype *Tg26*/+; *Pod::Cre/+*; *Myh9^flox^*/+×*Myh9^flox/flox^*.

Cohort of littermates	Genotype	Expected frequency
“TKO”	*Tg26/+; Pod::Cre/+*; *Myh9^flox^*/*^flox^*	1/8
“THet”	*Tg26/+; Pod::Cre/+*; *Myh9^flox^*/*^+^*	1/8
“Tg26”ff	Tg26/+; +/+ (cre−) ; *Myh9^flox^*/*^flox^*	1/8
“Tg26”	Tg26/+; +/+ (cre−) ; *Myh9^flox^*/*^+^*	1/8
Not used	*Pod::Cre/+*; *Myh9^flox^*/*^flox^*	1/8
Not used	*Pod::Cre/+*; *Myh9^flox^*/*^+^*	1/8
Not used	+/+ (cre−) ; *Myh9^flox^*/*^flox^*	1/8
Not used	+/+ (cre−) ; *Myh9^flox^*/*^+^*	1/8

d each parental allele was backcrossed to C57BL/6- the *Tg26* transgene 7 times, and the *Myh9^flox^* allele more than 10 times.

**Figure 3:**
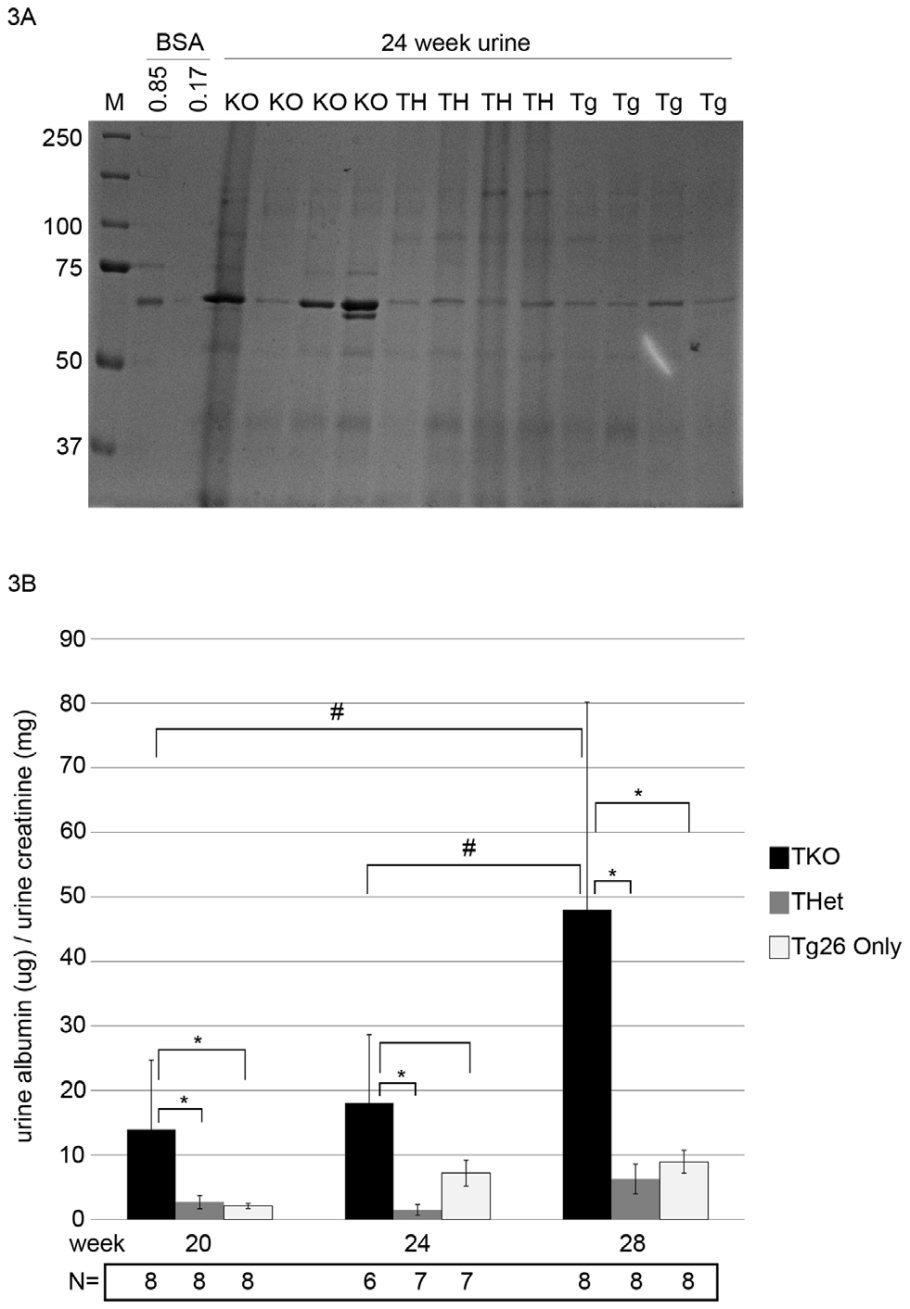
PodΔ***Myh9*** **results in hypersensitivity to HIV nephropathy from transgene** ***Tg26*** **.** (A) SDS-PAGE gel with coomassie stain of urine from 24 week old mice from littermates of Tg26+ crosses as outlined in Table 2. TKO = triple knockout, TH = triple heterozygous control, and Tg = Tg26/+; f/+ “only” genotype. M = molecular weight marker, and BSA are lanes of albumin standards in ug per lane. The TKO urines have more albuminuria, although the results are variable and not normalized to urine concentration with creatinine. (B) Quantification of albuminuria from mouse urine at weeks 20–28 of the indicated genotypes assessed by plate ELISA and normalized to urine creatinine. Yaxis = the ratio of urine albumin to urine creatinine (ug/mg). Beneath the abscissa, N = number of mice assessed for each genotype and each timepoint. Two findings are of statistical significance. First, within each month, the TKO mice have a significantly higher urine ACR than control littermates (*); for 20 weeks, TKO vs. TH (p = 0.0349), and TKO vs. Tg (p = 0.0277); for 24 weeks, TKO vs. TH (p = 0.0127), while the TKO vs. Tg showed a trend (p = 0.0575); for 28 weeks, TKO vs. TH (p = 0.0080) and TKO vs. Tg 0.0109). Secondly, the urine ACR in TKO mice increases over time (#) such that there is a significant difference between the 20 and 28 week TKO (p = 0.019), and a significant difference between the 24 and 28 week TKO (p = 0.0390). Statistical analyses employed unpaired t-test without the assumption of equal variances (Welch correction).

**Figure 4:**
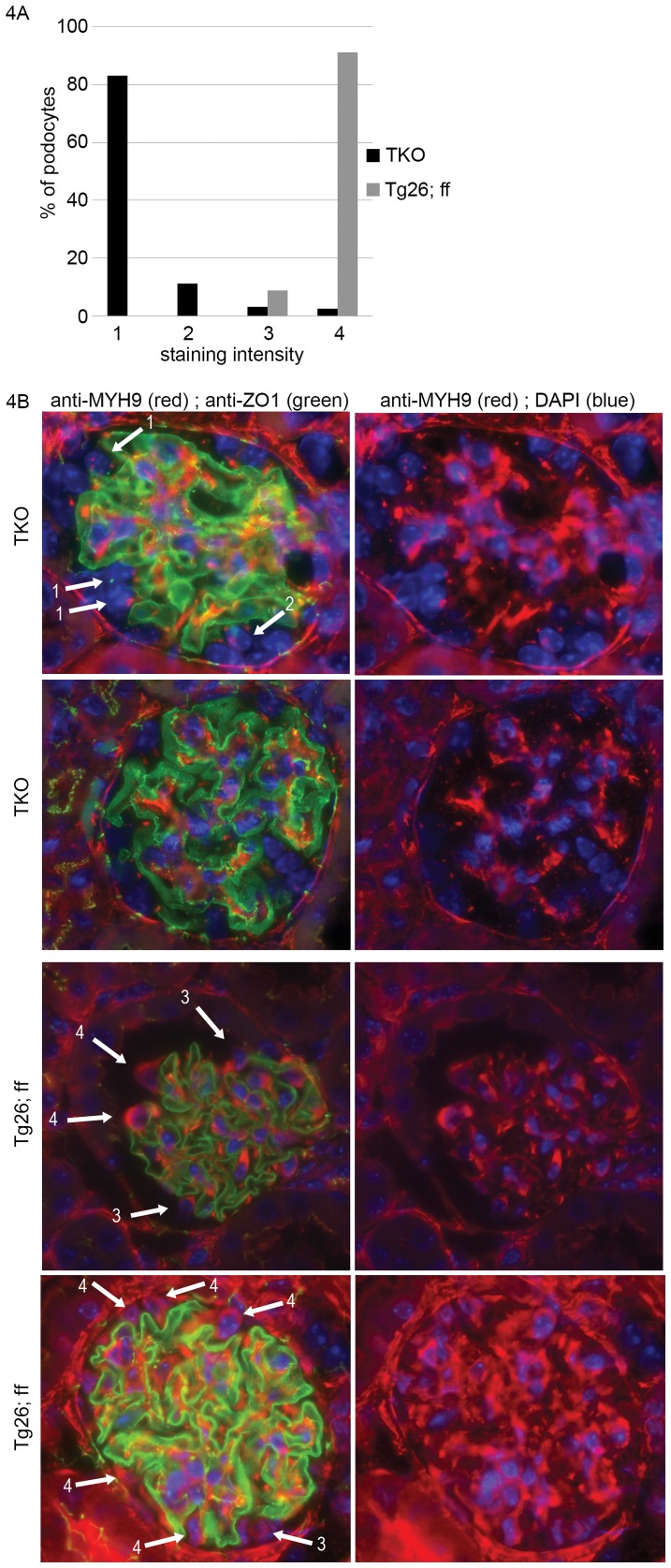
Confirmation of PodΔ***Myh9*** **in the presence of transgene** ***Tg26*** **.** MYH9 immunofluorescence was performed on kidney sections from triple knockout TKO mice and control littermates. Sections were processed in parallel from deparafinization through mounting to reduce staining variability and were stained with a mixture of 2 primary antibodies followed by a mixture of 2 secondary antibodies as described in Fig. 2. (A) Staining of 154 podocytes from 56 glomeruli of 2 TKO (*Tg26*/+; *Podocin::Cre*/+; *Myh9^flox^*/*^flox^*) were compared with 80 podocytes from 18 glomeruli of control “*Tg26*/+; cre−; *Myh9^flox^*/*^flox^*” littermates. MYH9 staining intensity was scored subjectively by two reviewers as with Fig. 2: (1) = no staining, (2) = faint/equivocal staining, (3) = moderate/incomplete staining, (4) = robust staining. (B) Examples of glomeruli stained for MYH9 and ZO1 used in the tally in part A, with photos taken at 630× magnification. Almost all podocytes of TKO mice were scored as “1” (no staining) and almost all podocytes of control (*Tg26*/+; cre−; *Myh9^flox^*/*^flox^*) mice were scored as a “4” (robust staining). By non-parametric rank-sum analysis, there was a significant difference between MYH9 podocyte staining of TKO versus control mice (P<0.0001 by Mann-Whitney test). No difference in ZO-1 staining was observed between TKO and control mice, suggesting consistency in fixation, deparafination, and staining.

At initial timepoints, there was no difference in albuminuria between TKO mice and either THet-control or Tg26-control mice as assessed both by Coomassie screening and albumin ELISA (all p>0.1). With aging through 28 weeks, we found a modest but significantly higher albuminuria in TKO mice compared to both Tg26+ control groups at 20 through 28 weeks (Fig. 3). To verify Cre-mediated excision of *Myh9* in podocytes in the presence of Tg26, we compared immunofluoresence of kidneys from TKO mice compared to *Tg26*/+; +/+; *Myh9^flox/flox^* control mice. Similar to Fig. 2, we found a nearly complete absence of cytoplasmic staining of MYH9 in TKO mouse podocytes (Fig. 4), consistent with efficient Cre-mediated excision of *Myh9*, and with minimal evidence of mosaicism at the level of immunodetection. We conclude that, in response to the Tg26 transgene backcrossed to C57BL/6 for seven generations, PodΔ*Myh9* results in a modest but significant predisposition to HIV-nephropathy in mature mice.

As an additional model of experimental glomerular injury, we examined whether PodΔ*Myh9* results in a predisposition to injury from sheep nephrotoxic serum (NTS), which includes an acute, complement dependent phase and a subsequent “autologous” phase of glomerular injury that is dependent on FcR [27]. While the mechanisms of acute NTS are more completely characterized than the later autologous phases, the acute NTS model of glomerular injury remains complex due to evidence of multiple targets in the glomerulus including decay accelerating factor [28], CD59 [29], and others [30]–[32]. Using mice backcrossed over 10× to C57BL/6, we injected PodΔ*Myh9* (KO) mice versus DHet control and FF control littermates with sheep NTS (graciously provided by Dr. D. Salant) or with control sheep IgG. Genotypes of parents and offspring were identical to Table 1 with the exception that all mice were at least 10× backcrossed to the C57BL/6 strain rather than FVB/N.

**Figure 5:**
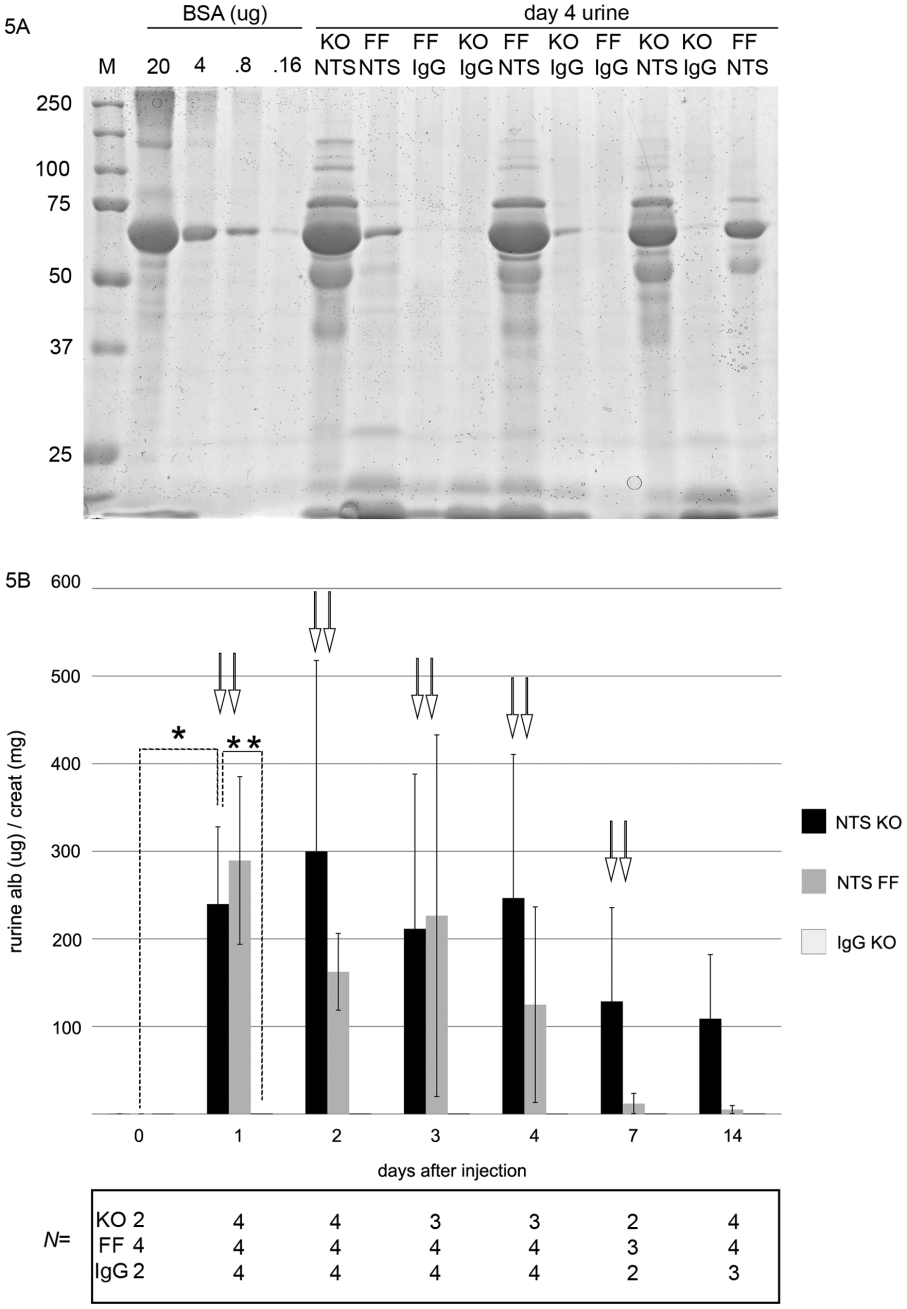
PodΔ***Myh9*** **mice (KO) are not hypersensitive to acute glomerular injury from sheep nephrotoxic serum (NTS).** C57BL/6 mice between 12 and 24 weeks of age were tested for predisposition to glomerular injury after injection of either 2.5 mg of sheep NTS or 2.5 mg of control sheep IgG, each resuspended in sterile phosphate buffered saline. Urine was collected at t = 0 before injection, then at days 1, 2, 3, 4, 7, and 14 after injection. (A) SDS-PAGE with Coomassie stain of urine 4 days after injection to screen for albuminuria. M = marker (Precision Plus Dual Color, Bio-Rad); BSA = bovine serum albumin in ug/lane. Sample lanes of the indicated genotype contain 2.5 microliters of urine in sample buffer. (KO) = *Podocin::Cre/+; Myh9^flox/flox^* and (FF) = *cre−; Myh9^flox/flox^*. (B) Quantification of albuminuria normalized to urine concentration. Y axis is the ACR (ratio of albuminuria to urine creatinine), similar to Figures 1 and 3. Numbers of animals for each timepoint and each genotype are indicated below the abscissa. Data were analyzed by unpaired T-test with Welch correction. On one hand, as intended, KO mice had significantly more albuminuria after receiving NTS: (_*_) ACR on day1 after NTS was 239+/−44 ug/mg versus day 0 ACR of 0.2 ug/mg+/−0.0007, p = 0.01, and albuminuria after NTS injection persisted through day 14. Similarly (**), the ACR on day1 after NTS was 239+/−44 ug/mg versus 0.4+/−0.3 ug/mg on day 1 after control sheep IgG, p = .012). However, (↓↓) for each day after injection with NTS, there is no difference in the magnitude of albuminuria between KO and control mice: day 1 p = 0.58; day 2 p = 0.31; day 3 p = 0.20; day 4 p = 0.396; day 7 p = 0.37. At day 14, there is a trend towards a difference between KO and control mice injected with NTS, p = 0.06, but in addition to the low *N*, this late timepoint is confounded by the contribution of an Fc dependent response from the immune system.

We performed pilot studies in order to determine a submaximal dose of NTS that would cause modest albuminuria in control animals and thereby allow detection of hypersensitivity in PodΔ*Myh9* animals. Mice on the C57BL/6 background are resistant to NTS and require a dose of 6.6 mg for full effect (D. Salant, pers comm). Doses in our pilot study ranged from 6 mg down to 0.375 mg (2–3 animals per dose). We found no overt albuminuria with 0.375 mg, moderate albuminuria and no deaths with 1.5–2.5 mg, and severe albuminuria with systemic illness necessitating euthanasia in 2 animals within 1 week after a dose of 6.6 mg. Based on these results, using a block design, PodΔ*Myh9* or control mice (DHets or *cre−*; *Myh9^flox/flox^*) were injected with either 2.5 mg NTS or 2.5 mg control sheep IgG, each reconstituted in sterile PBS. Compared to control genotypes we found that PodΔ*Myh9* (labeled KO for lack of space) were not hypersensitive to the acute phase of NTS (Fig. 5). At later time points, PodΔ*Myh9* mice appeared to have significantly more albuminuria than control mice, but this phase of injury includes FcR-dependent mechanisms of injury and will require additional analysis: because *Myh9* is the only conventional myosin heavy chain expressed in most of the immune system (including T-cells), we cannot say whether the effects on the later phase of NTS injury were secondary to podocyte-specific deletion of *Myh9* or to unintended Cre-mediated excision of *Myh9* from immune cells (or both). In contrast to the predisposition to glomerulosclerosis in response to either Adriamycin or the *Tg26* transgene, we conclude that PodΔ*Myh9* does not result in a predisposition to injury from the acute phase of sheep NTS.

For additional models we chose puromycin aminonucleoside, which causes a strain-specific podocytopathy to which C57BL/6 mice are resistant [22], [33]. In a pilot study, puromycin aminonucleoside or saline was injected IP at doses of 100, 250 or 500 mg/kg into 2 anesthetized mice of each of 3 genotypes (KO, DHet and FF control). Urine was collected at day 0, 3, 6, 9, 14, 21 and 28. No significant albuminuria was detected in any genotype at all doses and at all timepoints (Fig. S3). In a subsequent trial a higher dose of either 750 mg/kg or 1000 mg/kg of puromycin aminonucleoside was injected IP into 2 KO mice of each genotype and urine was collected at days 0, 3, 7, and 14. No significant albuminuria was detected by Coomassie screen and the protocol was complicated by >50% death before the final urine collection in all genotypes at 1000 mg/kg, suggesting this is a maximal dose for puromycin.

## Discussion

*MYH9* encoding myosin heavy chain 2A is one of three paralogs of conventional myosin heavy chain in mammals. As described in the Introduction, mutations in human *MYH9* cause a rare, autosomal dominant Giant Platelet syndrome with variably expressive but severe glomerulosclerosis that progresses to end stage kidney disease in young adults. In addition, human polymorphisms in *MYH9* correlate with several common kidney diseases including primary focal and segmental glomerulosclerosis and hypertensive nephrosclerosis, although the causality and mechanism for these common kidney diseases remains unclear. We previously hypothesized that disruption of *MYH9* function results in glomerular disease due to podocyte dysfunction. This hypothesis is based on the expression of *MYH9* in podocytes and because *MYH9* functions in aspects cytoskeletal dynamics that are believed to be critical to normal podocyte biology [34] including the establishment of cell shape, the maintenance of cell membrane tensile strength, regulation of cell-cell adhesion via focal adhesion complex interactions with the cytoskeleton, and rearrangement of the cytoskeleton during cell movement [18]. However, very little is known about the mechanisms by which *MYH9* mutations cause kidney disease *in vivo* in rare Giant Platelet syndromes and possibly in more common forms of kidney disease.

In previous work we reported that a podocyte-specific deletion of *Myh9* in mice (PodΔ*Myh9*) on the C57BL/6 strain background had no overt phenotype but instead resulted in a predisposition to glomerulosclerosis in response to a second injury from Adriamycin [3]. Other colleagues reported that PodΔ*Myh9* in mice can result in spontaneous glomerulosclerosis, as roughly 30% of knockout mice in their cohort developed severe albuminuria glomerulosclerosis on a mixed background of 3 strains (C57BL/6, BALB/c, and 129/SJ) [19] (and Dr. R.S. Adelstein, personal communication). We hypothesized, similar to work on the genetic basis of strain-dependent phenotypes of Adriamycin nephropathy [20] and HIV nephropathy in mice [21], that congenic crosses between strains resistant to and strains sensitive to PodΔ*Myh9* could identify genetic loci responsible for this strain-sensitive glomerulosclerosis due to PodΔ*Myh9*. In turn, this might provide key insight into the mechanisms of *Myh9*-related disease in humans, as analysis of clinical data from affected families with autosomal dominant *Myh9* disease reveals that adults have an all-or-nothing phenotype of kidney disease, either proteinuria with end stage kidney disease as young adults, or no proteinuria with normal renal function, all members of the family having similarly severe platelet defects and the identical autosomal dominant mutation. Such variable penetrance for *Myh9*-related kidney disease was suggested to result from the influence of additional modifying genes [3], which might be uncovered through congenic analysis of resistant and sensitive mouse strains.

This study used a combination of speed congenics and traditional backcrossing of the *Podocin::Cre* and *Myh9^flox^* alleles to achieve over 99% FVB/N, a background that is generally sensitive to diverse models of experimental glomerular disease including Adriamycin, HIV-nephropathy, and tetraspannin mutations as described in the Introduction. However, we were unable to find a spontaneous phenotype of glomerulosclerosis of PodΔ*Myh9* on the FVB/N background, even after aging mice to 28 weeks. In discussion with our colleagues from NIH, the explanation for our different observations remains difficult to prove, but we have a favored hypothesis. First, there are several explanations that are possible but unlikely. It is possible that a residual resistance factor from the C57BL/6 strain persisted after backcrossing. While our FVB/N strain was not fully backcrossed, the N6 cohort was over 99% FVB/N at the sixth generation due to combined speed congenics and traditional backcrossing, and we saw no outliers (no single animals with severe proteinuria) suggestive of the variable expressivity or reduced penetrance that is often seen with incomplete backcrossing. It is formally possible (but unlikely) that a resistance locus is closely linked to the insertion site of the *Podocin::Cre* transgene or to the *Myh9^flox^* allele itself, and in the process of selecting animals at each generation of backcrossing that are positive for *Pod::Cre* and *Myh9^flox^* we were selecting against the FVB strain at these two loci. Other possibilities include genetic differences in our separately generated *Myh9^flox^* alleles (discussed below), differences in the pathogen exposure of our mouse colonies, or a combinatorial effect from the 3 strains used by our colleagues (the % contribution of each strain in mice that did or did not have proteinuria cannot be determined).

However, we suspect that the explanation is related to the *Pod::Cre* transgene. Cre transgenes can exert deleterious effects in two general ways: either by high Cre activity that can act on pseudo-LoxP sites elsewhere in the genome of the target cell, or by a position effect of the insertion site of the transgene. In some instances the effects of non-specific Cre toxicity have been sufficient to explain experimental results of major studies. For instance, candidate genes for jeuvenile diabetes that relied on the RIP::Cre transgene were questioned subsequent to the finding of glucose intolerance in RIP::Cre mice themselves due to Cre-mediated toxicity of islet cells. Similarly, studies of hematopoetic development and differentiation that used two lines of transgenic Cre mice were questioned by the finding of direct toxicity of the Cre transgene on thymic development and hematopoeisis [35], [36]. To control for this, our crosses generated the PodΔ*Myh9* and control genotypes with a single copy of the *Pod::Cre* transgene (Table 1). Instead, if two double heterozygous animals are intercrossed as performed by Zhang and colleagues [19] (R. Adelstein, pers comm) then a different spectrum of genotypes is expected (see Table 3); among mice that were genotyped as KO animals, 1/3 would have been homozygous for the *Pod::Cre* transgene and 2/3 would have been heterozygous. We suspect (but cannot prove) that the *Pod*::*Cre* homozygous mice (*Pod::Cre/Pod::Cre*; *Myh9^flox^/Myh9^flox^*) were at risk either for a recessive effect at the transgene insertion site, or for podocyte toxicity from high levels of Cre recombinase.

**Table 3:** Expected genotypes of PodΔ*Myh9* and littermates as generated by Zhang and colleagues from two double heterozygous parents^b^ of genotype *Pod::Cre/+*; *Myh9^flox^*/+×*Pod::Cre/+*; *Myh9^flox^*/+.

Cohort of littermates	Genotype	Expected frequency
“KO” or PodΔ*Myh9*	c *Pod::Cre/Pod::Cre*; *Myh9^flox^*/*^flox^*	1/16 (1/3 of all KO)
“KO” or PodΔ*Myh9*	*Pod::Cre/+*; *Myh9^flox^*/*^flox^*	2/16 (2/3 of all KO)
“DHet” not tested	c *Pod::Cre/Pod::Cre*; *Myh9^flox^*/*^+^*	2/16
“DHet” not tested	*Pod::Cre/+*; *Myh9^flox^*/*^+^*	4/16
“FF” control	+/+ (cre−) ; *Myh9^flox^*/*^flox^*	1/16
Not used	Other combinations	6/16

b each parent was on a mixed background of 3 strains as described in the text.

c genotype is homozygous for the Pod::Cre transgene.

Other genetic differences between the separately generated *Myh9* floxed alleles should be considered. For example, if the allele created by Leon and colleages, which we used, undergoes Cre-mediated at a lower efficiency, this could explain why we found no phenotype in PodΔ*Myh9* mice. A rigorous comparison of the two *Myh9* floxed alleles would involve creating double mutant mice using the same promoter::Cre transgene, fully backcrossing these double mutants to the same background strain, and performing this analysis in the same mouse colony to control for environmental effects including pathogens. However, this rigorous comparison has not been performed and in its absence a comparison of the two floxed alleles rests on other data.

The first floxed allele, created by Leon and colleagues, targeted *loxP* sites around the exon containing the initiator ATG. They called this “exon 1” but subsequent analysis as summarized in NCBI and Ensembl demonstrates that exon 1 is a 5′ UTR that was not recognized by the authors at the time they designed their strategy, so to make a semantic clarification, these authors targeted exon2. The main question is whether deletion of exon 2 provides a convincing null allele, or whether exon 1 could splice to exon 3 or other downstream exons and then create a functional MYH9 polypeptide via a downstream ATG initiator. We focused on alternative transcripts that begin with exon 1 splicing to exon 3 because the motor domain arises from the 5′ end of the transcript, so alternative splicing to exon 4 or further downstream begins to delete the motor domain and would be unlikely to result in a functional protein. As shown in Fig. S4, we deleted exon 2 from the *Myh9* transcript and searched for downstream in-frame initiator ATGs. The process of translation initiation is still not fully understood and for this reason there are multiple algorithms for predicting translation initiation sites; as experts caution, none of these algorithms are excellent and some are terrible. In a careful analysis of available algorithms for predicting translation initiation sites, researchers compared the performance of multiple commercial and academic algorithms using a large series of genes with fairly detailed information on transcript variants and polypeptide variants and found that the program “ATGpr” performed best [37]. Analyzing the exon-2 deleted *Myh9* transcript with the “ATGpr” algorithm, which is available online by its creators [38], revealed no candidate sites with a high likelihood of rescuing function (Figure S4). The highest “reliability score,” which roughly equates to the chance that a true transcript is found or that a false transcript is not identified, was 0.65 for a transcript of 885 amino acids. However, this polypeptide would certainly delete the motor domain. Motor-defective polypeptides are used by researchers in the kinesin and myosin fields as dominant negatives [39], suggesting that N-terminal truncated MYH9 polypeptides, if produced, would cause dominant negative effects rather than provide rescue of function. The ATPpr algorithm identified one internal ATG that would created a nearly full length transcript (1824 amino acids, compared to 1960 full length). However, this site had a lower “reliability score” of 0.56, a doublet was not observed in the immunoblots of Leon et al, and we suspect that normal protein function would not tolerate deletion of 136 amino acids from the N-terminal motor domain. In C. elegans there are two alleles of the ortholog of *Myh9* that involve amino acid substitutions of this same region (allele *gk411521* at amino acid 59 and *gk645135* at amino acid 45 of *Nmy-2*). Overall, this *in silico* analysis suggests that “escape” from the knockout strategy of Leon and colleagues is theoretically possible but unlikely, and if anything should make the phenotype more severe, whereas we observed no phenotype in mice after aging to 9 months (Fig. 1).

The second conditional allele of *Myh9*, created by Zhang and colleagues, targeted *loxP* sites around exon 3. The question is whether this provides a convincing null allele, or whether alternative splicing or the absence of an in-frame deletion could result in escape from flox-mediated deletion or other alternative polypeptides. These possibilities are extremely unlikely. First, there is no evidence that exon 2 can undergo alternative splicing to exon 4 from available transcripts in NCBI or Ensembl. Second, deletion of the 157 nucleotides of exon 3 would result in a frameshift when exon 2 splices to exon 4. Translation of this exon 3-deleted transcript (Fig. S4) demonstrates that this also includes nonsense mutations 55 and 61 codons after the frameshift. These early nonsense codons should result in degradation of the entire transcript due to the process of nonsense-mediated decay. In summary, this is a convincing molecular null allele. *In vivo*, quantification of deletion efficiency for this allele resulted in MYH9 protein levels that were 18% of normal (Jacobelli et al, Fig. S2 from Jacobelli et al [40]) in comparison with the “less than 3% of normal” reported by Leon and colleagues. However, Jacobelli and colleagues assessed *Myh9* deletion with a modified *Lck* promoter to express Cre recombinase in T-cells [40], whereas Leon and colleagues used the PFA4::Cre transgene in Platelets. Variable Cre expression by different transgenes has a major impact on deletion efficiency at *loxP* sites. Until both *Myh9* conditional alleles are compared with the same Cre-transgene, no definitive conclusions can be made about the relative efficiency of deletion of the two *loxP* alleles.

A rigorous comparison of the two conditional *Myh9* alleles should utilize Cre expression in a tissue for which *Myh9* dysfunction results in a clear phenotype. For several reasons, the Platelet Factor 4::Cre transgene used by Leon and colleagues would serve as a good tool to compare these conditional alleles. To begin with, *Myh9* mutations result in a strong phenotype in platelets (hence the “Giant Platelet Syndrome” as the defining and most consistent phenotype of human *Myh9* mutations). In part, the strong phenotype in platelets arises because platelets do not express paralogs of *Myh9* such as *Myh10* encoding myosin heavy chain 2B (podocytes also do not express *Myh10* [*3*] but other candidate genes have not been fully assessed). In addition, a pool of platelets can be purified with comparative ease from a blood sample such that MYH9 protein levels can be quantified from purified platelet lysate. When Leon and colleagues did this using immunoblots they estimated that residual MYH9 protein in their platelet knockout mice was “less than 3% of normal.” This could represent a low level of ongoing gene expression in platelets, but it is more likely that this “less than 3%” corresponds to MYH9 protein that was translated prior to Cre-mediated deletion of the floxed allele. The PFA4::Cre transgene begins to express Cre recombinase when Megakaryocyte/platelet development begins, whereas *Myh9* begins expression much earlier in the single celled zygote. Accordingly, one expects some transcription and translation of *Myh9* prior to Cre-mediated deletion by *PFA4::Cre*. Another possibility is that this 3% of residual protein after platelet knockout represents mosaicism, in which case 3% of the platelet pool might express *Myh9* and be functionally normal. However, in the analysis by Leon and colleagues the platelets were all Giant-sized, rather than a subpopulation of 3% of platelets with normal size as one would expect from a low level of mosaicism. Accordingly, the floxed allele of Leon et al appeared to be capable of efficient excision. We believe that *Myh9* deletion was similarly efficient in our hands with the *Podocin::Cre* transgene, and while we could not assess this in purified podocyte lystates (mesangial cells and possibly endothelial cells express *Myh9* quite robustly), the deletion appeared robust based on the lack of podocyte staining by immunofluoresence as shown previously by Johnstone et al [3], and in Figures 2 and 4.

In summary, we hypothesize that the spontaneous phenotype of glomerulosclerosis seen among roughly 30% of PodΔ*Myh9* animals on a mixed background may correspond to those PodΔ*Myh9* animals that were homozygous for the *Pod::Cre* transgene. This hypothesis cannot be proven or disproven from the available data but is a cautionary note for future investigations.

The second question addressed in this study was whether podocyte-specific deletion of *Myh9* on the C57BL/6 background results in predisposition to glomerulosclerosis in general, perhaps from a “weakened” podocyte, or whether the predisposition is specific to Adriamycin injury. We found, similar to Adriamycin, that PodΔ*Myh9* resulted in a predisposition to glomerulosclerosis due to the Tg26 transgenic model of HIV-nephropathy, including a higher level of albuminuria by quantitative ELISA. In a previous report, investigators found that loss of *Myh9* did not predispose mice to *Tg26*-nephropathy [14], but our results are not contradictory. This previous report used a different experimental design in which Tg26/+ mice were compared to Tg26/+ mice that were heterozygous for a classical null allele of *Myh9*. None of the mice were homozygous KO animals, as this classical null allele of *Myh9* results in early embryonic lethality when homozygous. The absence of an effect on Tg26 nephropathy from heterozygous loss of *Myh9* in this previous study is consistent with the reports that mice with one copy of this null allele had either a very subtle phenotype of sensorineural hearing loss or no phenotype at all, leading to the conclusion that the *Myh9* locus is not haploinsufficient [41], [42]. A similar conclusion on the lack of haploinsufficiency in humans can be inferred from the spectrum of mutations found among those with autosomal dominant *MYH9*-related disease: there are scores of distinct missense mutations but no nonsense mutations (except in the last exon, in which nonsense mediated decay has little influence) [1]. The absence of canonical nonsense mutations is indirect evidence that the *MYH9* locus in humans is not haploinsufficient. In mice, we conclude that PodΔ*Myh9* predisposes to experimental nephropathy due to both Adriamycin and the *Tg26* model of HIV-nephropathy, and this predisposition requires more than the loss of one *Myh9* allele.

In contrast to Adriamycin and the *Tg26* model of HIV nephropathy, we found no evidence of a predisposition due to PodΔ*Myh9* from other models of injury including puromycin aminonucleoside and sheep nephrotoxic serum (NTS). It is possible that hypersensitivity could be uncovered in PodΔ*Myh9* mice at a different dose of sheep NTS, but based on our pilot study, we chose a dose that appeared intermediate and should have allowed for detection of an effect. At this time it is unclear why podocyte-specific deletion of *Myh9* exerts a greater sensitivity to some models of injury as compared to others. Unfortunately, we have an incomplete understanding of the mechanisms underlying most models of experimental glomerular injury as well as an incomplete understanding of the role of *Myh9* in response to these models. Future studies will aim to uncover the mechanisms by which *Myh9* functions in kidney disease.

## Materials and Methods

Ethics statement: Mice were raised and all experiments using mice were performed in accordance with protocol 803389 approved by the Institutional Animal Care and Use Committee of the University of Pennsylvania (multiple project assurance #A3079-01). Mouse strain generation and genotyping: *Podocin:: Cre*/+; *Myh9^flox/flox^* (PodΔ*Myh9*) mice on the C57BL/6 background (>10×backcrossed) were generated as previously described (Johnstone et al, 2011) with additional backcrossing to C57BL/6 (JAX strain 664) as needed to expand desired genotypes. *Podocin::Cre*/+; *Myh9^flox/flox^* (PodΔ*Myh9*) mice on the FVB background were generated by backcrossing to FVB/N (Jax strain 1800). At generations 2, 3 and 4, mouse tail DNA was submitted for speed congenics to Charles River (Max-Bax) to select progeny with the greatest % FVB based on PCR amplification of multiple strain-specific polymorphisms interspersed approximately 7 Mbp apart on each chromosome. The N4 founder mouse, 93.625% FVB and shaded yellow (Fig. S1), was backcrossed for 2 more generations yielding a predicted 99.3125% FVB/N at N6). Among N6 progeny, *Myh9^flox^* animals were intercrossed to make an N6 *Myh9^flox/flox^* stock, which were crossed with N6 DHeterozygous animals in experimental crosses as outlined in Table 1. Concurrent to these experiments, mice were additionally backcrossed and N10 animals will be submitted to the MMRRC. Allele *Tg26* was acquired on a mixed background for improved chances of survival during transport and quarantine. After backcrossing the *Tg26* allele 7 times to C57BL/6 resulting in an estimated 98.4375% BL/6, we generated “triple knockout mice” on the C57BL/6 background (*Tg26*/+; *Podocin::Cre*/+; *Myh9^flox/flox^*) by crossing triple heterozygous males X *Myh9^flox/flox^* females as outlined in Table 2.

Genotyping was performed on mouse tail DNA digested overnight at 60°C in DirectPCR reagent (Viagen) with 10% v/v 1 mg/mL Proteinase K solution, followed by heat inactivation for 15 minutes at 92–94°C. PCR reactions used GoTaq Green 2× Master Mix (Thermo Scientific) or DreamTaq 2× Master Mix (Promega). For the *Tg26* allele, we used primers that identified a linked, flanking polymorphism (TGTCAATTTCACGGACAATGCT) and primers to HIV within the Tg26 transgene that avoided the LTRs (GGGAGGGTGATTGTGTCACTCC). The *Podocin::Cre* allele and the *Myh9^flox^* allele were genotyped as previously described (Johnstone et al, 2011). All PCR reactions included 2 sets of primers (4 primers total), one set to either transgene (band present or absent) and one set to *Myh9* (band always present, size differs), such that the absence of either Cre or Tg26 in a given lane always included a positive internal control for effective PCR based on the *Myh9* locus. All PCR reactions were aliquotted from a master mix of GoTaq with 2 sets of PCR primers freshly added.

Urine albumin to creatinine ratio was calculated from clean-catch urine at 4-week timepoints beginning at 4 weeks of age as described previously (Johnstone et al. 2011). Briefly, mice were placed in cages atop elevated wire mesh for 2–4 hours. Urine free of contaminating feces was collected and spun at 5000 g for 10 minutes in a tabletop centrifuge, and the supernatant was frozen at −20°C or −80°C until assayed. Urine was qualitatively screened for albuminuria by SDS-PAGE and Coomassie staining as described previously (Johnstone et al. 2011). For quantitative albumin::creatinine ratios, albuminuria was determined by plate ELISA (albuwell-M kit from Glycadia/Exocell). Each plate included 16 wells (8 concentrations in duplicate) to generate a standard curve, and all samples were tested in duplicate, with data used only if the duplicate results were within the linear range of the standard curve and if the two duplicate samples were <10% different. Calculations of standard curve and of sample concentrations were performed using Excel (Microsoft). Urine creatinine was determined by endpoint assay (TECO diagnostics) in microtitre plates, using 16 wells (8 concentrations in duplicate) to generate a standard curve from a creatinine stock solution of 1 mg/mL (Sigma). Albumin and creatinine microtitre plates were read using a multiplate reader (Beckman-Coulter DTX 880).

For microscopy, kidneys were prepared as described previously (Johnstone et al. 2011). Briefly, mice were perfused via the infrarenal aorta with 37°C warmed Hank's buffered salt solution×3–5 minutes at 90 mmHg under a closed system with compressed air, then perfused with 4% w/v paraformaldehyde-PBS solution×3–5 minutes, then gently shaken in 4% paraformaldehyde-PBS overnight (not more than 24 hrs). After fixation, tissue was dehydrated using serial PBS and ethanol washes and submitted to the Abramson Cancer Center Histology Core (University of Pennsylvania) for paraffin embedding and sectioning. For immunofluoresence, 4 micron sections were deparaffinized using xylene/ethanol (per ABCAM). Antigen retrieval conditions were optimized by block design for different actual thickness of sections, with preferred conditions for heat retrieval of 10 mM Tris, 1 mM EDTA pH = 9.0 for 2 hours at 95°C. Antibody incubation and sample mounting were performed as described previously (Johnstone et al. 2011). We also observed decreased background of MYH9 staining after purifying the crude serum with Melon Gel (Pierce). Photos were obtained with an Axio Observer.D1m microscope (Zeiss) at 200× and 630× using Metamorph software (Molecular Devices), and for all immunoflourescent pictures, we removed the automated exposures to keep the time and contrast settings equal between samples we wished to compare. After blocking, α-ZO1 from Zymed and α-MYH9 12874 rabbit polyclonal (Johnstone et al. 2011) were diluted in Tris-buffered saline+1% BSA+0.025% TritonX-100, washed 6 times with the same Tris buffer, blocked again with Tris buffer+10%goat serum+1%BSA+0.025% TritonX100, then incubated with 1∶1500 goat α-mouse488 and goat α-rabbit594 or goat α-rabbit568 AlexaFluor antibodies (Molecular Probes/Invitrogen).

Several models of experimental glomerular disease were employed. For sheep nephrotoxic serum, mice were anesthetized by intraperitoneal injection of 15 uL/gram of sterile 2.5% Avertin in phosphate buffered saline, then injected retro-orbitally with 2.5 mg of sheep nephrotoxic serum or 2.5 mg of control sheep IgG. All injections appeared fully absorbed and all mice recovered without complication. For puromycin aminonucleoside, there is no single protocol in current use for mice. In one study [33] authors found that C57BL/6 mice are resistant to 100 mg/kg but with 500 mg/kg SQ they found hypersensitivity in mice with G-protein coupled receptor mutation, with a caveat that this SQ protocol was complicated by up to 20% mortality. Another study used a lower dose of 18.25 mg/100 gram mouse but with 2 retro-orbital injections [22], and no mention is made of death or complications. Puromycin aminonucleoside is best described for rats [43], in which a 150 mg/kg dose by IP injection results in dramatic proteinuria after 3 days. Accordingly, we opted for a single intraperitoneal dose of puromycin amononucleoside (100 mg/kg, 250 mg/kg, 500 mg/kg, or PBS-only), followed by a higher dose pilot with 750 and 1000 mg/kg.

## Supporting Information

Figure S1 **Results of speed congenics after 4 generations of backcrossing to FVB/N.** The founder mouse for subsequent crosses is #950 (shaded). Given space, the full spreadsheet showing results from chromosomes 1–14 is not shown.
(PDF)

Figure S2 **SDS-PAGE of mouse urine with Coomasie stain to screen for albuminuria in experimental crosses that included the** ***Tg26*** **transgenic model of HIV nephropathy.** Mice were 6–8 weeks old at the time of urine collection. MW = molecular weight marker, Precision Plus (Bio-Rad). BSA = standards of bovine serum albumin, either 2 µg per lane or 0.2 µg per lane. Remaining lanes are samples of mouse urine from 6–8 week old mice of the indicated genotypes (lanes 6–7 and 11–12 were 6 weeks old). Neg = a PodΔ*Myh9* littermate from the experimental crosses in Table 1 that was negative for *Tg26*. In all samples, low molecular weight protein is abundant in the urine but very little albuminuria is visible.
(PDF)

Figure S3 **SDS-PAGE of urine from littermates on a pure C57BL/6 background following challenge with puromycin aminonucleoside.** Albumin standards were loaded on each gel (BSA in µg/lane). Each urine lane is labeled with the mouse genotype (KO, DHet control, FF control) and type of injection (saline, or puromycin in mg/kg). Samples were loaded by tag#, blinded to genotype and injection. Day 0 urine samples were loaded in a different order into lanes and also showed no albuminuria. One gel (group2, day 6) is missing the last lane due to fecal contamination of the urine sample on that day.
(PDF)

Figure S4 **Work sheet documenting the *in silico* comparison of the two *loxP* conditional alleles of *Myh9***.
(PDF)
