# Supplementary material for: Background Strain and the Differential Susceptibility of Podocyte-Specific Deletion of Myh9 on Murine Models of Experimental Glomerulosclerosis and HIV Nephropathy
Source: PLoS One. 2013 Jul 10;8(7):e67839. doi: 10.1371/journal.pone.0067839 (PMC3707882; doi:10.1371/journal.pone.0067839)
Supplement: Figure S1 — Results of speed congenics after 4 generations of backcrossing to FVB/N. The founder mouse for subsequent crosses is #950 (shaded). Given space, the full spreadsheet showing results from chromosomes 1–14 is not shown. (PDF) [file pone.0067839.s001.pdf]

Supplementary Fig S1: generation 4 speed congenics

1 = both FVB
0 = both BL/6
0.5 = 1 of each

Chr1 (SNPs 1-8) through Chr15 (SNPs 1-3) not shown

mouse tag #

Marker ID	CM	M#	930	939	940	944	950	962	967	968	971	974	975	976	980
Chr15-5	57.4	**81**	1	1	1	1	1	0.5	0.5	0.5	1	0.5	0.5	0.5	0.5
Chr16-1	22.2	**82**	1	1	1	1	1	1	0.5	1	1	0.5	0.5	1	1
Chr16-2	43.1	**83**	1	1	1	1	1	1	0.5	0.5	1	0.5	0.5	1	1
Chr16-3	57.6	**84**	0.5	0.5	0.5	1	0.5	1	0.5	0.5	1	0.5	0.5	0.5	0.5
Chr17-1	18.8	**85**	1	1	1	1	1	1	1	1	1	1	1	1	1
Chr17-2	22.9	**86**	1	1	1	1	1	1	1	1	1	1	1	1	1
Chr17-3	29.5	**87**	0.5	1	1	1	1	1	1	1	1	1	1	1	1
Chr17-4	44.5	**88**	0.5	1	1	1	1	1	1	1	1	1	1	1	1
Chr18-1	20.0	**89**	1	1	1	1	1	1	1	1	1	1	1	1	1
Chr18-2	37.0	**90**	1	1	1	1	1	1	1	1	1	1	1	1	1
Chr18-3	45.0	**91**	1	1	1	1	1	1	1	1	1	1	1	1	1
Chr18-4	50.0	**92**	1	1	1	1	1	1	1	1	1	1	1	1	1
Chr19-1	6.0	**93**	1	1	1	1	1	1	1	1	1	1	1	1	1
Chr19-2	15.0	**94**	1	1	1	1	1	1	1	1	1	1	1	1	1
Chr19-3	24.0	**95**	1	1	1	1	1	1	1	1	1	1	1	1	1
Chr19-5	47.0	**96**	1	1	1	1	1	1	1	1	1	1	1	1	1
ChrX-1	17.3	**97**	1	1	1	1	1	1	1	1	1	1	1	1	1
ChrX-2	33.0	**98**	1	1	1	1	1	1	1	1	1	1	1	1	1
ChrX-3	43.0	**99**	1	1	1	1	1	1	1	1	1	1	1	1	1
ChrX-4	60.0	**100**	1	1	1	1	1	1	1	1	1	1	1	1	1
ChrX-5	70.0	**101**	1	1	1	1	1	1	1	1	1	1	1	1	1
**Results= %FVB**			**92.50**	**94.50**	**94.06**	**93.56**	**96.53**	**94.55**	**92.57**	**93.50**	**94.06**	**91.58**	**92.50**	**90.59**	**93.07**
							BEST								

Figure legend for supplementary Fig S1: Results of speed congenics after 4 generations of backcrossing to FVB/N. The founder mouse for subsequent crosses is #950 (shaded). Given space, the full spreadsheet showing results from chromosomes 1-14 is not shown.
