# Supplementary material for: Background Strain and the Differential Susceptibility of Podocyte-Specific Deletion of Myh9 on Murine Models of Experimental Glomerulosclerosis and HIV Nephropathy
Source: PLoS One. 2013 Jul 10;8(7):e67839. doi: 10.1371/journal.pone.0067839 (PMC3707882; doi:10.1371/journal.pone.0067839)
Supplement: Figure S2 — SDS-PAGE of mouse urine with Coomasie stain to screen for albuminuria in experimental crosses that included the Tg26 transgenic model of HIV nephropathy. Mice were 6–8 weeks old at the time of urine collection. MW = molecular weight marker, Precision Plus (Bio-Rad). BSA = standards of bovine serum albumin, either 2 µg per lane or 0.2 µg per lane. Remaining lanes are samples of mouse urine from 6–8 week old mice of the indicated genotypes (lanes 6–7 and 11–12 were 6 weeks old). Neg = a PodΔMyh9 littermate from the experimental crosses in Table 1 that was negative for Tg26. In all samples, low molecular weight protein is abundant in the urine but very little albuminuria is visible. (PDF) [file pone.0067839.s002.pdf]

Supplementary Fig S2

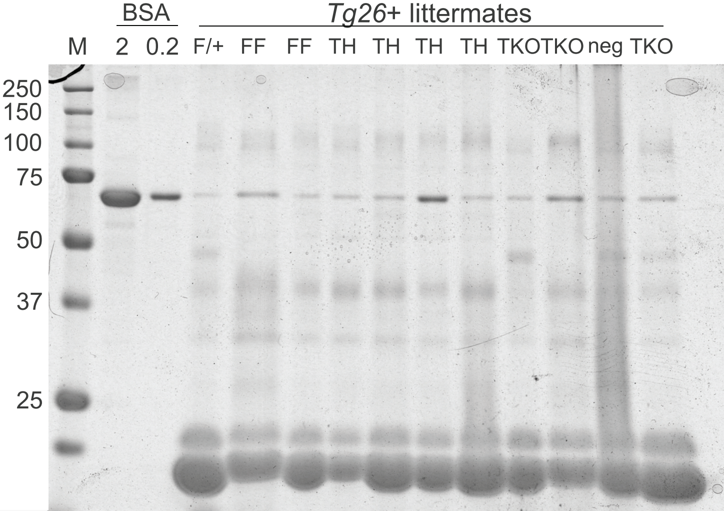

Figure legend for supplementary Fig S2: SDS-PAGE of mouse urine with Coomasie stain to screen for albuminuria in experimental crosses that included the *Tg26* transgenic model of HIV nephropathy. Mice were 6-8 weeks old at the time of urine collection. MW = molecular weight marker, Precision Plus (Bio-Rad). BSA= standards of bovine serum albumin, either 2ug per lane or 0.2ug per lane. Remaining lanes are samples of mouse urine from 6-8 week old mice of the indicated genotypes (lanes 6-7 and 11-12 were 6 weeks old). Neg= a PodΔ*Myh9* littermate from the experimental crosses in Table 1 that was negative for *Tg26*. In all samples, low molecular weight protein is abundant in the urine but very little albuminuria is visible.
