# Supplementary material for: Background Strain and the Differential Susceptibility of Podocyte-Specific Deletion of Myh9 on Murine Models of Experimental Glomerulosclerosis and HIV Nephropathy
Source: PLoS One. 2013 Jul 10;8(7):e67839. doi: 10.1371/journal.pone.0067839 (PMC3707882; doi:10.1371/journal.pone.0067839)
Supplement: Figure S3 — SDS-PAGE of urine from littermates on a pure C57BL/6 background following challenge with puromycin aminonucleoside. Albumin standards were loaded on each gel (BSA in µg/lane). Each urine lane is labeled with the mouse genotype (KO, DHet control, FF control) and type of injection (saline, or puromycin in mg/kg). Samples were loaded by tag#, blinded to genotype and injection. Day 0 urine samples were loaded in a different order into lanes and also showed no albuminuria. One gel (group2, day 6) is missing the last lane due to fecal contamination of the urine sample on that day. (PDF) [file pone.0067839.s003.pdf]

Supp Fig S3: susceptibility to puromycin aminonucleoside

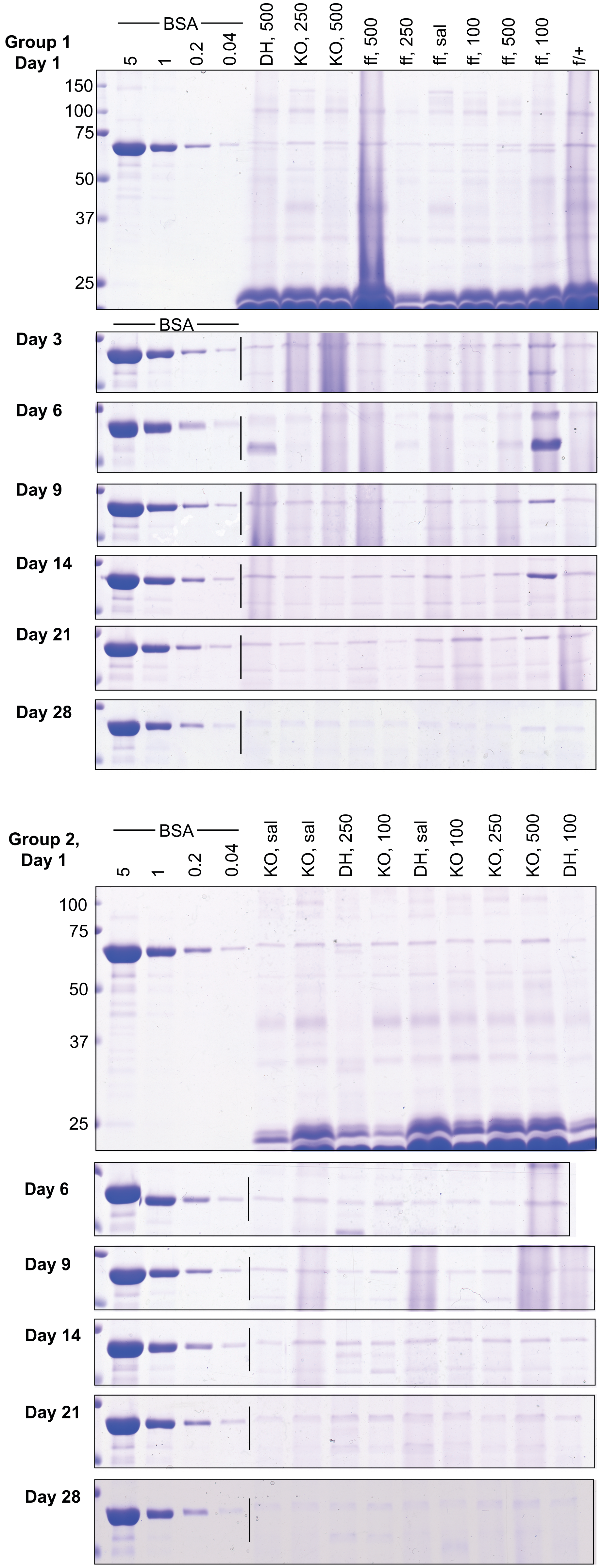

Figure legend for supplementary Fig S3: SDS-PAGE of urine from littermates on a pure C57BL/6 background following challenge with puromycin aminonucleoside. Albumin standards were loaded on each gel (BSA in ug/lane). Each urine lane is labeled with the mouse genotype (KO, DHet control, FF control) and type of injection (saline, or puromycin in mg/kg). Samples were loaded by tag#, blinded to genotype and injection. Day 0 urine samples were loaded in a different order into lanes and also showed no albuminuria. One gel (group2, day 6) is missing the last lane due to fecal contamination of the urine sample on that day.
