# Supplementary material for: Background Strain and the Differential Susceptibility of Podocyte-Specific Deletion of Myh9 on Murine Models of Experimental Glomerulosclerosis and HIV Nephropathy
Source: PLoS One. 2013 Jul 10;8(7):e67839. doi: 10.1371/journal.pone.0067839 (PMC3707882; doi:10.1371/journal.pone.0067839)
Supplement: Figure S4 — Work sheet documenting the in silico comparison of the two loxP conditional alleles of Myh9. (PDF) [file pone.0067839.s004.pdf]

Supplementary Fig S4: worksheet for comparison of the two conditional flox alleles of Myh9:

1) Leon and colleagues deleted exon 2 containing the ATG.

From Ensembl, exon 2 includes a bit of UTR followed by the initiator ATG (in BOLD and separated by a space):
GTCCTGGCCGCAAGTCACC **ATG**GCTCAGCAGGCTGCAGACAAGTACCTCTATGTGGATAA
AAACTTCATCAATAACCCGCTGGCCCAAGCTGACTGGGCTGCCAAGAAGTTGGTATGGGT
GCCTTCCAGCAAGAATGGCTTTGAACCAGCTAGCCTCAAGGAGGAGGTGGGAGAAGAGGC
CATTGTAGAGCTGGTAGAGAATGGGAAGAAGGTGAAGGTGAACAAGGACGACATCCAGAA
GATGAACCCACCCAAGTTCTCCAAGGTGGAGGACATGGCAGAGCTCACGTGCCTCAACGA
AGCTTCGGTGCTGCACAACCTCAAGGAGCGATACTACTCAGGGCTTATCTAC

Assuming deletion of exon 2, could exon 1 splice to exon 3 and then create a polypeptide via a downstream ATG initiator?

Starting with the full transcript of Myh9, shown below, exon 2 is crossed out, and the remaining transcript from exon3 onwards was analyzed by available algorithms to look for alternative ATG initiation sites.

>gi|114326445|ref|NM_022410.2| Mus musculus myosin, heavy polypeptide 9, non-muscle (Myh9), transcript variant 1, mRNA
AGATCACCGCGGTTCCTGGGCAGGGCACGGAAGGCTCAAGAACCTGACCTGCTGCAGCTTCCAGTCTCGC
GTTCGCCCCACCCCGCCGCGCCGCCCGAGCGCTCGAGAAAGTCCACTCGGAAGAACCAGCGCCTGTTCCC
CGGGCAGACCCAGGTTCAG~~GTCCTGGCCGCAAGTCACCATGGCTCAGCAGGCTGCAGACAAGTACCTCTA~~
~~TGTGGATAAAAACTTCATCAATAACCCGCTGGCCCAAGCTGACTGGGCTGCCAAGAAGTTGGTATGGGTG~~
~~CCTTCCAGCAAGAATGGCTTTGAACCAGCTAGCCTCAAGGAGGAGGTGGGAGAAGAGGCCATTGTAGAGC~~
~~TGGTAGAGAATGGGAAGAAGGTGAAGGTGAACAAGGACGACATCCAGAAGATGAACCCACCCAAGTTCTC~~
~~CAAGGTGGAGGACATGGCAGAGCTCACGTGCCTCAACGAAGCTTCGGTGCTGCACAACCTCAAGGAGCGA~~
~~TACTACTCAGGGCTTATCTAC~~
ACCTATTCAGGCCTGTTCTGTGTGGTCATCAACCCTTATAAGAACCTGC
CCATCTACTCAGAGGAGATCGTGGAGATGTACAAGGGCAAGAAGAGGCACGAGATGCCACCCCACATCTA
CGCCATCACAGATACTGCCTACCGGAGCATGATGCAGGACCGGGAAGATCAGTCCATCCTGTGCACGGGG
GAGTCTGGAGCAGGGAAGACAGAGAACACCAAGAAAGTCATCCAGTACCTGGCACATGTGGCCTCCTCAC

ACAAGAGCAAGAAGGACCAGGGGGAGTTGGAGCGGCAGCTGCTACAGGCCAACCCTATCCTAGAGGCCTT
TGGAAACGCCAAGACGGTGAAGAATGACAACTCCTCTCGATTCGGTAAATTCATTCGTATCAACTTTGAT
GTCAATGGCTACATTGTTGGTGCCAACATTGAGACTTATCTTCTGGAGAAATCTCGTGCTATCCGCCAAG
CCAAAGAGGAGCGGACCTTCCACATCTTCTACTACCTGCTGTCTGGGGCCGGAGAACACCTGAAGACTGA
TCTCCTGTTGGAGCCATACAACAAATACCGCTTCCTGTCCAACGGGCACGTCACCATCCCTGGGCAGCAG
GACAAGGACATGTTCCAGGAGACAATGGAGGCCATGAGAATTATGGGCATCCCAGAGGATGAGCAGATGG
GCTTGCTGCGGGTCATCTCTGGGGTCCTTCAGCTTGGCAACATTGCCTTCAAGAAGGAGCGGAACACTGA
CCAGGCGTCCATGCCGGACAACACAGCTGCTCAAAAGGTGTCCCACCTCCTGGGGATCAATGTGACCGAC
TTCACCAGAGGCATCCTCACCCCACGCATCAAGGTGGGCAGAGACTATGTGCAGAAGGCGCAGACTAAAG
AGCAGGCTGACTTTGCCATTGAGGCCTTGGCCAAGGCTACCTATGAGCGGATGTTCCGCTGGCTGGTGCT
TCGCATCAACAAAGCTCTGGACAAGACCAAGAGGCAGGGCGCCTCATTTATCGGGATCCTGGACATCGCT
GGCTTTGAGATCTTTGATCTGAACTCCTTCGAGCAGCTGTGCATCAACTACACCAACGAGAAGCTGCAGC
AGCTGTTCAACCACACCATGTTCATCCTGGAGCAGGAGGAGTACCAGCGAGAGGGCATCGAGTGGAACTT
CATCGACTTCGGCCTGGACCTGCAGCCCTGCATCGACCTCATTGAGAAGCCGGCGGGTCCCCCAGGCATC
CTGGCCCTGCTAGATGAGGAGTGCTGGTTTCCTAAGGCCACTGACAAGAGCTTCGTGGAGAAGGTGGTGC
AGGAGCAGGGCACCCACCCCAAGTTCCAGAAGCCCAAGCAACTGAAGGACAAGGCTGATTTCTGCATTAT
CCACTATGCCGGCAAGGTGGACTATAAAGCTGACGAGTGGCTGATGAAGAACATGGACCCCTTGAACGAC
AACATCGCCACGCTGCTTCACCAGTCCTCAGACAAGTTTGTCTCTGAGCTGTGGAAGGATGTGGATCGGA
TCATTGGCTTGGACCAAGTGGCTGGAATGTCCGAGACAGCACTACCTGGTGCCTTCAAGACCCGGAAGGG
CATGTTCCGTACTGTCGGACAGCTGTACAAGGAGCAGCTGGCCAAGCTCATGGCCACGTTGAGGAATACC
AACCCCAACTTCGTGCGCTGCATCATTCCCAACCATGAGAAGAAGGCCGGCAAACTGGACCCGCACTTGG
TGCTGGACCAGCTGCGCTGCAATGGCGTCCTTGAGGGCATCCGGATCTGCCGCCAGGGCTTTCCCAACAG
GGTGGTCTTCCAGGAGTTCCGGCAGAGGTATGAGATCCTCACCCCCAACTCCATCCCGAAGGGCTTCATG
GATGGCAAGCAAGCGTGTGTGCTCATGATCAAAGCCTTGGAGCTTGACAGCAACCTGTACCGCATCGGCC
AGAGCAAAGTGTTCTTCCGGGCAGGAGTGCTAGCCCACCTGGAGGAAGAGCGGGACCTGAAGATCACCGA
TGTCATCATTGGCTTCCAGGCCTGCTGCAGGGGCTACCTGGCCAGGAAGGCCTTTGCCAAGAGGCAGCAA
CAGCTGACCGCCATGAAGGTCCTACAGAGGAACTGTGCTGCGTACCTCAGGCTGCGCAACTGGCAGTGGT
GGAGGCTCTTCACCAAGGTCAAGCCCCTGTTGAACTCAATAAGACATGAGGATGAGCTGTTAGCCAAGGA
GGCGGAACTGACAAAGGTTCGAGAGAAACATCTGGCTGCAGAGAACAGGCTGACAGAGATGGAGACGATG

CAGTCTCAGCTCATGGCAGAGAAGCTGCAGCTTCAGGAGCAGCTGCAGGCGGAGACAGAGCTGTGTGCCG
AGGCTGAGGAGCTCCGGGCCCGTCTGACAGCGAAGAAGCAGGAGCTGGAGGAGATCTGCCATGACCTGGA
GGCCAGGGTGGAGGAGGAGGAGGAGCGCTGCCAGTACCTGCAGGCCGAGAAGAAGAAGATGCAGCAGAAC
ATCCAGGAACTTGAGGAGCAGTTGGAGGAGGAGGAGAGCGCCCGGCAGAAGCTGCAGCTTGAGAAGGTGA
CCACCGAGGCCAAGCTGAAGAAACTGGAGGAGGACCAGATCATCATGGAGGACCAGAACTGCAAACTGGC
CAAGGAGAAGAAACTGCTGGAAGACAGAGTAGCTGAATTCACTACCAACCTCATGGAAGAGGAGGAGAAG
TCCAAGAGCCTGGCCAAGCTCAAGAACAAGCACGAGGCAATGATCACCGACCTGGAAGAGCGCCTCCGTA
GGGAGGAGAAGCAGAGGCAGGAGTTGGAGAAGACCCGTCGCAAGCTGGAGGGAGACTCCACAGACCTCAG
TGACCAGATTGCTGAGCTCCAGGCGCAGATAGCAGAGCTCAAGATGCAGCTGGCCAAGAAGGAGGAGGAG
TTGCAGGCTGCCTTGGCCAGAGTGGAAGAAGAAGCTGCTCAGAAGAATATGGCCCTGAAGAAGATCCGAG
AACTGGAAACTCAGATCTCTGAGCTCCAGGAGGACCTGGAGTCGGAGCGAGCCTCCAGGAATAAAGCCGA
GAAGCAGAAACGGGATCTGGGAGAGGAGCTGGAGGCGCTGAAGACAGAGCTGGAGGACACGCTGGACTCC
ACGGCTGCCCAGCAGGAGCTGAGGTCGAAGCGTGAGCAGGAGGTGAGCATCCTGAAGAAGACTCTGGAGG
ACGAGGCCAAGACCCATGAGGCCCAGATCCAGGAGATGAGGCAGAAGCACTCACAGGCTGTGGAGGAGCT
GGCAGATCAGTTGGAGCAGACGAAGCGGGTAAAAGCTACCCTTGAGAAGGCGAAGCAGACCCTGGAGAAT
GAGCGGGGAGAGCTGGCCAATGAGGTGAAGGCCCTGCTGCAAGGCAAGGGCGACTCAGAGCACAAGCGCA
AGAAGGTGGAGGCGCAGCTGCAAGAACTGCAGGTCAAGTTCAGCGAGGGAGAGCGCGTGCGAACCGAACT
GGCCGACAAGGTCACCAAGCTGCAGGTTGAACTGGACAGTGTGACCGGTCTCCTTAGCCAGTCTGACAGC
AAGTCCAGCAAGCTTACGAAGGACTTCTCTGCGCTGGAGTCCCAGCTTCAGGACACACAGGAGTTGCTCC
AGGAGGAGAACCGGCAGAAGCTGAGCCTGAGCACCAAGCTCAAGCAGATGGAGGATGAGAAAAACTCCTT
CAGGGAGCAGCTGGAGGAGGAGGAGGAGGCCAAGCGCAACTTGGAGAAGCAGATCGCCACGCTCCATGCC
CAGGTGACCGACATGAAGAAGAAGATGGAGGACGGTGTAGGGTGCCTGGAGACTGCAGAGGAGGCGAAGC
GGAGGCTTCAGAAGGACTTGGAAGGCCTGAGCCAGCGGCTTGAGGAGAAGGTGGCTGCCTACGATAAGCT
GGAGAAGACCAAGACACGGCTGCAGCAGGAGCTGGACGACCTGCTGGTTGACCTGGACCACCAGCGGCAG
AGCGTCTCCAACCTGGAAAAGAAGCAGAAGAAGTTCGACCAGCTCCTAGCCGAGGAGAAGACCATCTCGG
CCAAGTATGCAGAGGAGCGTGACCGAGCTGAGGCTGAGGCCCGTGAGAAGGAGACAAAGGCGCTATCACT
GGCCCGGGCGCTTGAGGAGGCCATGGAGCAGAAGGCAGAGCTGGAGCGGCTCAACAAGCAGTTCCGCACG
GAGATGGAGGACCTCATGAGCTCCAAGGATGACGTGGGCAAGAGTGTCCACGAGCTGGAGAAGTCCAAGC
GGGCCTTGGAGCAGCAGGTGGAGGAGATGAAGACCCAGCTGGAGGAGCTGGAGGATGAGCTGCAGGCCAC

GGAGGATGCCAAGCTCCGCCTGGAGGTGAACCTGCAGGCCATGAAGGCCCAGTTTGAGCGGGATCTGCAG
GGCCGGGATGAACAGAGCGAGGAGAAGAAGAAGCAGCTGGTCAGACAGGTGCGGGAGATGGAGGCGGAGC
TGGAGGATGAGAGGAAGCAGCGCTCCATGGCCATGGCCGCACGCAAGAAACTGGAGATGGATCTGAAGGA
CCTGGAGGCACACATTGACACAGCCAATAAGAACCGGGAAGAGGCCATCAAACAGCTGCGGAAGCTTCAG
GCCCAGATGAAGGACTGCATGCGGGAGCTGGACGACACGCGCGCCTCCCGGGAGGAGATCCTGGCGCAGG
CCAAGGAGAATGAGAAGAAGCTGAAGAGCATGGAGGCCGAGATGATTCAGCTGCAGGAGGAACTGGCAGC
TGCTGAGCGTGCTAAGCGTCAGGCCCAACAGGAACGGGACGAGCTGGCTGATGAGATCGCCAACAGCAGT
GGCAAAGGGGCCCTAGCATTAGAGGAGAAGCGGCGACTGGAGGCCCGCATTGCCCAGCTGGAGGAGGAGC
TGGAGGAGGAACAGGGCAACACGGAGCTGATCAACGATCGGCTGAAGAAGGCCAACCTGCAGATCGACCA
AATAAACACCGACCTGAACCTGGAACGCAGCCACGCACAGAAGAATGAGAATGCGCGACAGCAGCTGGAA
CGCCAGAACAAGGAGCTCAAGGCCAAGCTGCAGGAAATGGAGAGTGCTGTCAAGTCCAAATACAAGGCCT
CCATCGCGGCCTTGGAGGCCAAAATTGCACAGCTGGAGGAACAGCTGGACAACGAGACCAAGGAGCGCCA
GGCAGCCTCCAAGCAGGTGCGCCGGACGGAGAAGAAGCTGAAGGACGTGCTGCTGCAGGTGGAGGACGAG
CGGAGGAACGCGGAACAGTTCAAGGACCAGGCTGACAAGGCGTCCACCCGCCTGAAGCAGCTTAAACGGC
AGCTAGAGGAGGCTGAAGAGGAGGCCCAGCGGGCCAATGCCTCACGCCGGAAGCTGCAGCGTGAGCTGGA
AGATGCCACAGAGACCGCTGATGCTATGAACCGCGAGGTCAGCTCCCTGAAGAACAAACTGAGGCGTGGG
GACCTGCCATTTGTCGTGACTCGCCGAATTGTTCGGAAAGGCACTGGCGACTGCTCAGACGAGGAGGTCG
ACGGTAAAGCAGATGGGGCCGATGCCAAGGCAGCTGAATAGGAGCTTCTCCTGCAGCCCAGGCGGATGGA
CAAACGGCTCTGCCTCCCTCCCCCAACCCTCCACACCCCTGCCTTGAGACTGCTCTGACCATGTCCCCCT
CCTCCCAAGGCCTTCCCGAGGGCATTGGCTTCCTCTGCTGCAGCCCTTCCAGTCCTCCATACCCTTTGAG
AATCTGATACCAAAGAGTCCAGGCTGGCTCAGGCCGGATGACCCACAGGGTCTTGTCCTCCTTGCCTGAA
AGCACGGGTGGTGGGCAAGAAGGGCGGCCATTGGAGTAGGCACAAGAGTTTTCTATGAATCTATTTTGTC
TTCAGATAAAGATTTTGATAGCTCAGGCCTCTAGTAGTGTTACCCTCCCCGACCTCGGCTGTCCCCGTCC
CCCGTCCCCCCTGCTGTTGGCAATCACACACGGTAACCTCATACCTGCCCTATGGCCCCCTTCCCTGGGC
CCTATTGGTCCAGAAGGAGCCTCTGTGCTGGGTGCAGAACATGGGGCACTCTGGGAATCCCCCCACTCCC
TTCTGGGCAGCACTGGTGCCTCTGCTCCTCCGACTGTAAACCGTCTCAAGTGCAATGCCCCTCCCCTCCC
TTGCCAAGGACAGACCGTCCTGGCACCGGGGCAAACCAGACAGGGCATCAGGGCCACTCTAGAAAGGCCA
ACAGCCTTCCGGTGGCTTCTCCCAGCACTCTAGGGGACCAAATATATTTAATGGTTAAGGGACTTGCAGG
GCCTGGCAGCCAGAATATCCAAGGGCTGGAGCCCACTGTGCGCTCTGGTGCCTCTCCTAGGACTGGGGCC

AAGGGTGGTCGAGCTGTGCCACCCACTCTATAGCTTCAAGTCTGCCTTCCACAAGGATGCTTTTGAAAGA
AAAAAAAAGGTTTTATTTTTCCCTTCTTGTAGTAAGTGCTCTAGTTCTGGGTGTCTTCACTGCCTTGCCC
TGGAACTGTGTTTAGAAGAGAGTAGCTTGCCCTACAATGTCTACACTGGTCGCTGAGTTCCCTGCGCACT
GCACCTCACTGTTTGTAAATGCTGTGATTAGGTTCCCTTATGGCAGGAAGGCTTTTTTTTTCTTTTTTTT
TTTCTTTTCTTTTTTTTTTTTTTAAAGGAAAACCAGTCAAATCATGAAGCCACATACGCTAGAGAAGCTG
AATCCAGGTCCCAAAGGCGCTGTCATAAAGGAGCAAGTGGGACCCGCACCCCTTTTTTTATATAATACAA
GTGCCTTAGCATGTGTCGCAGCTGTCACCACTACAGTAAGCTGGTTTACAGATGTTTCCACTGAGCGTCA
CAATAAAGAGTACCATGTCCT

What is the best way to find a downstream alternative initiator ATG?

There are several algorithms for translation initiation, but it is known that none are excellent, and some are terrible (because we do not fully understand what governs translation initiation yet). In 2004, a group of researchers evaluated multiple programs in parallel and felt the program “ATGpr” performed best in side-by-side testing using a series of genes with fairly detailed information on transcript variants and polypeptide variants. “Comparison of computational methods for identifying translation initiation sites in EST data” Afshin Nadershahi[1], Scott C Fahrenkrug[2] and Lynda BM Ellis[3]* BMC Bioinformatics 2004, 5:14 doi:10.1186/1471-2105-5-14.”

Accordingly, I ran the exon-2 deleted transcript of Myh9 through the “ATGpr” algorithm, which is available online and was created by Salamov AA, Nishikawa T, Swindells MB: Assessing protein coding region integrity in cDNA sequencing projects. Bioinformatics 1998, 14:384-390. The “ATGpr” algorithm identified, as shown in the table below, one nearly full length transcript (1824 amino acids, compared to 1960 full length) but with a fairly low “reliability score” of 0.56, suggesting it is unclear whether or not this could serve as an efficient alternative initiator ATG (equates to roughly a 56% chance that a true transcript is found or that a false transcript is not identified). The highest reliability score for alternate ATG sites was 0.65 for a transcript of 885 amino acids. However, this polypeptide would not include the motor domain, and such motor-defective polypeptides are used by researchers in the kinesin and myosin fields as dominant negatives. If anything, this should cause a severe podocyte dysfunction, whereas we found no phenotype in PodΔMyh9 mice on two strain backgrounds.

From “ATGpr”

No.of ATG from 5'end	Reliability	Frame	Identity to Kozak rule A/GXXATGG	Start (bp)	Finish (bp)	ORF Length (aa)	Stop codon found ?	Sequence
47	0.65	1	AXXATGc	2893	5547	885	Yes	MQLAKKEEELQAALARVEEEAAQKNMALKKIRELETQISELQEDLESERA<br>SRNKAEKQKRDLGEELEALKTELEDTLDSTAAQQELRSKREQEVSILKKT<br>LEDEAKTHEAQIQEMRQKHSQAVEELADQLEQTKRVKATLEKAKQTLENE<br>RGELANEVKALLQGKGDSEHKRKKVEAQLQELQVKFSEGERVRTELADK<br>V<br>TKLQVELDSVTGLLSQSDSKSSKLTKDFSALESQLQDTQELLQEENRQKL<br>SLSTKLKQMEDEKNSFREQLEEEEEAKRNLEKQIATLHAQVTDMKKKMED<br>GVGCLETAEEAKRRLQKDLEGLSQRLEEKVAAYDKLEKTKTRLQQELDDL<br>LVDLDHQRQSVSNLEKKQKKFDQLLAEEKTISAKYAEERDRAEAEAREKE<br>TKALSLARALEEAMEQKAELERLNKQFRTEMEDLMSSKDDVGKSVHELEK<br>SKRALEQQVEEMKTQLEELEDELQATEDAKLRLEVNLQAMKAQFERDLQ<br>G<br>RDEQSEEKKKQLVRQVREMEAELEDERKQRSMAMAARKKLEMDLKDLEA<br>H<br>IDTANKNREEAIKQLRKLQAQMKDCMRELDDTRASREEILAQAKENEKKL<br>KSMEAEMIQLQEELAAAERAKRQAQQERDELADEIANSSGKGALALEEKR<br>RLEARIAQLEEELEEEQGNTELINDRLKKANLQIDQINTDLNLERSHAQK<br>NENARQQLERQNKELKAKLQEMESAVKSKYKASIAALEAKIAQLEEQLDN<br>ETKERQAASKQVRRTEKKLKDVLLQVEDERRNAEQFKDQADKASTRLKQ<br>L<br>KRQLEEAEEEAQRANASRRKLQRELEDATETADAMNREVSSLKNKLRRG<br>D<br>LPFVVTRRIVRKGTGDCSDEEVDGKADGADAKAAE
1	0.56	1	GXXATGt	76	5547	1824	Yes	MYKGKKRHEMPPHIYAITDTAYRSMMQDREDQSILCTGESGAGKTENTKK<br>VIQYLAHVASSHKSKKDQGELERQLLQANPILEAFGNAKTVKNDNSSRFG<br>KFIRINFDVNGYIVGANIETYLLEKSRAIRQAKEERTFHIFYYLLSGAGE<br>HLKTDLLLEPYNKYRFLSNGHVTIPGQQDKDMFQETMEAMRIMGIPEDEQ<br>MGLLRVISGVLQLGNIAFKKERNTDQASMPDNTAAQKVSHLLGINVTDFT<br>RGILTPRIKVGRDYVQKAQTKEQADFAIEALAKATYERMFRWLVLRINKA<br>LDKTKRQGASFIGILDIAGFEIFDLNSFEQLCINYTNEKLQQLFNHTMFI<br>LEQEEYQREGIEWNFIDFGLDLQPCIDLIEKPAGPPGILALLDEECWFPK

								ATDKSFVEKVVQEQGTHPKFQKPKQLKDKADFCIIHYAGKVDYKADEWLM<br>KNMDPLNDNIATLLHQSSDKFVSELWKDVDRIIGLDQVAGMSETALPGAF<br>KTRKGMFRTVGQLYKEQLAKLMATLRNTNPNFVRCIIPNHEKKAGKLDPH<br>LVLDQLRCNGVLEGIRICRQGFPNRVVFQEFRQRYEILTPNSIPKGFMDG<br>KQACVLMIKALELDSNLYRIGQSKVFFRAGVLAHLEEERDLKITDVIIGF<br>QACCRGYLARKAFAKRQQQLTAMKVLQRNCAAYLRLRNWQWWRLFTKV<br>KP<br>LLNSIRHEDELLAKEAELTKVREKHLAAENRLTEMETMQSQLMAEKLQLQ<br>EQLQAETELCAEAEELRARLTAKKQELEEICHDLEARVEEEEERCQYLQA<br>EKKKMQQNIQELEEQLEEEESARQKLQLEKVTTEAKLKKLEEDQIIMEDQ<br>NCKLAKEKKLLEDRVAEFTTNLMEEEEKSKSLAKLKNKHEAMITDLEERL<br>RREEKQRQELEKTRRKLEGDSTDLSDQIAELQAQIAELKMQLAKKEEELQ<br>AALARVEEEAAQKNMALKKIRELETQISELQEDLESERASRNKAEKQKRD<br>LGEELEALKTELEDTLDSTAAQQELRSKREQEVSILKKTLEDEAKTHEAQ<br>IQEMRQKHSQAVEELADQLEQTKRVKATLEKAKQTLENERGELANEVKAL<br>LQGKGDSEHKRKKVEAQLQELQVKFSEGERVRTELADKVTKLQVELDSVT<br>GLLSQSDSKSSKLTKDFSALESQLQDTQELLQEENRQKLSLSTKLKQMED<br>EKNSFREQLEEEEEAKRNLEKQIATLHAQVTDMKKKMEDGVGCLETAEEA<br>KRRLQKDLEGLSQRLEEKVAAYDKLEKTKTRLQQELDDLLVDLDHQRQSV<br>SNLEKKQKKFDQLLAEEKTISAKYAEERDRAEAEAREKETKALSLARALE<br>EAMEQKAELERLNKQFRTEMEDLMSSKDDVGKSVHELEKSKRALEQQVE<br>E<br>MKTQLEELEDELQATEDAKLRLEVNLQAMKAQFERDLQGRDEQSEEKKK<br>Q<br>LVRQVREMEAELEDERKQRSMAMAARKKLEMDLKDLEAHIDTANKNREE<br>A<br>IKQLRKLQAQMKDCMRELDDTRASREEILAQAKENEKKLKSMEAEMIQLQ<br>EELAAAERAKRQAQQERDELADEIANSSGKGALALEEKRRLEARIAQLEE<br>ELEEEQGNTELINDRLKKANLQIDQINTDLNLERSHAQKNENARQQLERQ<br>NKELKAKLQEMESAVKSKYKASIAALEAKIAQLEEQLDNETKERQAASKQ<br>VRRTEKKLKDVLLQVEDERRNAEQFKDQADKASTRLKQLKRQLEEAEEEA<br>QRANASRRKLQRELEDATETADAMNREVSSLKNKLRRGDLPFVVTRRIVR<br>KGTGDCSDEEVDGKADGADAKAAE
59	0.53	1	GXXATGG	4132	5547	472	Yes	MEQKAELERLNKQFRTEMEDLMSSKDDVGKSVHELEKSKRALEQQVEEM<br>K<br>TQLEELEDELQATEDAKLRLEVNLQAMKAQFERDLQGRDEQSEEKKKQLV<br>RQVREMEAELEDERKQRSMAMAARKKLEMDLKDLEAHIDTANKNREEAIK<br>QLRKLQAQMKDCMRELDDTRASREEILAQAKENEKKLKSMEAEMIQLQEE<br>LAAAERAKRQAQQERDELADEIANSSGKGALALEEKRRLEARIAQLEEEL

								EEEQGNTELINDRLKKANLQIDQINTDLNLERSHAQKNENARQQLERQNK<br>ELKAKLQEMESAVKSKYKASIAALEAKIAQLEEQLDNETKERQAASKQVR<br>RTEKKLKDVLLQVEDERRNAEQFKDQADKASTRLKQLKRQLEEAEEEAQR<br>ANASRRKLQRELEDATETADAMNREVSSLKNKLRRGDLPFVVTRRIVRKG<br>TGDCSDEEVDGKADGADAKAAE
56	0.52	1	GXXATGa	377<br>2	5547	592	Yes	MKKKMEDGVGCLETAEEAKRRLQKDLEGLSQRLEEKVAAYDKLEKTKTRL<br>QQELDDLLVDLDHQRQSVSNLEKKQKKFDQLLAEEKTISAKYAEERDRAE<br>AEAREKETKALSLARALEEAMEQKAELERLNKQFRTEMEDLMSSKDDVGK<br>SVHELEKSKRALEQQVEEMKTQLEELEDELQATEDAKLRLEVNLQAMKAQ<br>FERDLQGRDEQSEEKKKQLVRQVREMEAELEDERKQRSMAMAARKKLE<br>MD<br>LKDLEAHIDTANKNREEAIKQLRKLQAQMKDCMRELDDTRASREEILAQA<br>KENEKKLKSMEAEMIQLQEELAAAERAKRQAQQERDELADEIANSSGKGA<br>LALEEKRRLEARIAQLEEELEEEQGNTELINDRLKKANLQIDQINTDLNL<br>ERSHAQKNENARQQLERQNKELKAKLQEMESAVKSKYKASIAALEAKIAQ<br>LEEQLDNETKERQAASKQVRRTEKKLKDVLLQVEDERRNAEQFKDQADK<br>A<br>STRLKQLKRQLEEAEEEAQRANASRRKLQRELEDATETADAMNREVSSLK<br>NKLRRGDLPFVVTRRIVRKGTGDCSDEEVDGKADGADAKAAE
36	0.48	1	GXXATGa	209<br>2	5547	1152	Yes	MKVLQRNCAAYLRLRNWQWWRLFTKVKPLLNSIRHEDELLAKEAELTKVR<br>EKHLAAENRLTEMETMQSQLMAEKLQLQEQLQAETELCAEAEELRARLTA<br>KKQELEEICHDLEARVEEEEERCQYLQAEKKKMQQNIQELEEQLEEEESA<br>RQKLQLEKVTTEAKLKKLEEDQIIMEDQNCKLAKEKKLLEDRVAEFTTNL<br>MEEEEKSKSLAKLKNKHEAMITDLEERLRREEKQRQELEKTRRKLEGDST<br>DLSDQIAELQAQIAELKMQLAKKEEELQAALARVEEEAAQKNMALKKIRE<br>LETQISELQEDLESERASRNKAEKQKRDLGEELEALKTELEDTLDSTAAQ<br>QELRSKREQEVSILKKTLEDEAKTHEAQIQEMRQKHSQAVEELADQLEQT<br>KRVKATLEKAKQTLENERGELANEVKALLQGKGDSEHKRKKVEAQLQELQ<br>VKFSEGERVRTELADKVTKLQVELDSVTGLLSQSDSKSSKLTKDFSALES<br>QLQDTQELLQEENRQKLSLSTKLKQMEDEKNSFREQLEEEEEAKRNLEKQ<br>IATLHAQVTDMKKKMEDGVGCLETAEEAKRRLQKDLEGLSQRLEEKVAAY<br>DKLEKTKTRLQQELDDLLVDLDHQRQSVSNLEKKQKKFDQLLAEEKTISA<br>KYAEERDRAEAEAREKETKALSLARALEEAMEQKAELERLNKQFRTEMED<br>LMSSKDDVGKSVHELEKSKRALEQQVEEMKTQLEELEDELQATEDAKLRL<br>EVNLQAMKAQFERDLQGRDEQSEEKKKQLVRQVREMEAELEDERKQRS<br>MA<br>MAARKKLEMDLKDLEAHIDTANKNREEAIKQLRKLQAQMKDCMRELDDTR<br>ASREEILAQAKENEKKLKSMEAEMIQLQEELAAAERAKRQAQQERDELAD<br>EIANSSGKGALALEEKRRLEARIAQLEEELEEEQGNTELINDRLKKANLQ

								IDQINTDLNLERSHAQKNENARQQLERQNKELKAKLQEMESAVKSKYKAS<br>IAALEAKIAQLEEQLDNETKERQAASKQVRRTEKKLKDVLLQVEDERRNA<br>EQFKDQADKASTRLKQLKRQLEEAEEEAQRANASRRKLQRELEDATETA<br>D<br>AMNREVSSLKNKLRRGDLPFVVTRRIVRKGTGDCSDEEVDGKADGADAK<br>A<br>AE

----

2) The second conditional allele, created by Adelstein's group (Zhang et al), targeted exon 3 for deletion with loxP sites. The question is whether this provides a convincing null allele, or whether alternative splicing or the absence of an in-frame deletion could result in escape from the deletion of this exon, or whether other gene products could be translated since the ATG containing exon 2 is still present. First, there is no evidence that exon 3 can be skipped, as there are no splice forms that jump from exon 2 to exon 4 in NCBI or Ensembl (there appear to be no alternative splice variants, as the one variant cDNA is simply a partial length). Second, it is highly likely that deletion of exon 3 would result in a convincing, canonical null, as exon 3 is 157 nt, which should result in a frameshift and an early nonsense codon. In turn, due to nonsense mediated decay, the early nonsense codons would likely lead to degradation of the transcript.

Exon 3 is:
ACCTATTCAGGCCTGTTCTGTGTGGTCATCAACCCTTATAAGAACCTGCCCATCTACTCAGAGGAGATCGTGGAGATG
TACAAGGGCAAGAAGAGGCACGAGATGCCACCCCACATCTACGCCATCACAGATACTGCCTACCGGAGCATGATGC
AGG

Assuming deletion of exon 3, I have spliced together exon 2 (starting with the ATG) with exons 4 and onwards.
ATGGCTCAGCAGGCTGCAGACAAGTACCTCTATGTGGATAA
AAACTTCATCAATAACCCGCTGGCCCAAGCTGACTGGGCTGCCAAGAAGTTGGTATGGGT
GCCTTCCAGCAAGAATGGCTTTGAACCAGCTAGCCTCAAGGAGGAGGTGGGAGAAGAGGC
CATTGTAGAGCTGGTAGAGAATGGGAAGAAGGTGAAGGTGAACAAGGACGACATCCAGAA
GATGAACCCACCCAAGTTCTCCAAGGTGGAGGACATGGCAGAGCTCACGTGCCTCAACGA

AGCTTCGGTGCTGCACAACCTCAAGGAGCGATACTACTCAGGGCTTATCTAC
ACCGGGAAGATCAGTCCATCCTGTGCAC
GGGGGAGTCTGGAGCAGGGAAGACAGAGAACACCAAGAAAGTCATCCAGTACCTGGCACA
TGTGGCCTCCTCACACAAGAGCAAGAAGGACCAG
GGGGAGTTGGAGCGGCAGCTGCTACAGGCCAACCCTATCCTAGAGGCCTTTGGAAACGCC
AAGACGGTGAAGAATGACAACTCCTCTCGATTC
GGTAAATTCATTCGTATCAACTTTGATGTCAATGGCTACATTGTTGGTGCCAACATTGAG
ACTT
ATCTTCTGGAGAAATCTCGTGCTATCCGCCAAGCCAAAGAGGAGCGGACCTTCCACATCT
TCTACTACCTGCTGTCTGGGGCCGGAGAACACCTGAAGA
CTGATCTCCTGTTGGAGCCATACAACAAATACCGCTTCCTGTCCAACGGGCACGTCACCA
TCCCTGGGCAGCAGGACAAGGACATGTTCCAGGAGACAATGGAGGCCATGAGAATTATGG
GCATCCCAGAGGATGAGCAGATGG

The predicted polypeptide from this transcript is show below.
Translation of exon 2, which is still intact, is shown larger, bold text. The switch to exon4 begins with amino acids TGK (a frameshift) and includes nonsense codons 55 and 61 codons later (and more nonsense codons still further).

>EMBOSS_001_1
MAQQAADKYLYVDKNFINNPLAQADWAAKKLVWVPSSKNGFEPASLKEEVGEEAIVELVE
NGKKVKVNKDDIQKMNPPKFSKVEDMAELTCLNEASVLHNLKERYYSGLIYTGKISPSCA
RGSLEQGRQRTPRKSSSTWHMWPPHTRARRTRGSWSGSCYRPTLS*RPLETPRR*RMTTP
LDSVNSFVSTLMSMATLLVPTLRLIFWRNLVLSAKPKRSGPSTSSTTCCLGPENT*RLIS
CWSHTTNTASCPTGTSPSLGSRTRTCSRRQWRP*ELWASQRMSRW

This is a convincing molecular null allele, not just due to the frameshift, but due to nonsense mediated decay.

Figure legend for supplementary Fig S4: work sheet documenting the *in silico* comparison of the two loxP conditional alleles of *Myh9*.
